# Inflammation pathways as therapeutic targets in angiotensin II induced atrial fibrillation

**DOI:** 10.3389/fphar.2025.1515864

**Published:** 2025-03-03

**Authors:** Ailin Hou, Dazhuo Shi, Hongbo Huang, Yuxuan Liu, Ying Zhang

**Affiliations:** ^1^ Cardiovascular Department, Xiyuan Hospital, China Academy of Chinese Medical Sciences, Beijing, China; ^2^ Graduate School of Beijing University of Chinese Medicine, Xiyuan Hospital, Beijing, China

**Keywords:** atrial fibrillation, inflammation, AngII, electrical remodelling, fibrosis, anti-inflammatory therapy

## Abstract

Atrial fibrillation (AF), a common cardiac arrhythmia, is associated with severe complications such as stroke and heart failure. Although the precise mechanisms underlying AF remain elusive, inflammation is acknowledged as a pivotal factor in its progression. Angiotensin II (AngII) is implicated in promoting atrial remodeling and inflammation. However, the exact pathways through which AngII exacerbates AF are still not fully defined. This study explores the key molecular mechanisms involved, including dysregulation of calcium ions, altered connexin expression, and activation of signaling pathways such as TGF-β, PI3K/AKT, MAPK, NF-κB/NLRP3, and Rac1/JAK/STAT3. These pathways are instrumental in contributing to atrial fibrosis, electrical remodeling, and increased susceptibility to AF. Ang II-induced inflammation disrupts ion channel function, resulting in structural and electrical remodeling of the atria and significantly elevating the risk of AF. Anti-inflammatory treatments such as RAAS inhibitors, colchicine, and statins have demonstrated potential in reducing the incidence of AF, although clinical outcomes are inconsistent. This manuscript underscores the link between AngII-induced inflammation and the development of AF, proposing the importance of targeting inflammation in the management of AF.

## 1 Introduction

Atrial fibrillation (AF) is one of the most common persistent arrhythmias encountered in clinical practice. It is strongly linked to conditions such as hypertension, coronary artery disease, and heart failure, all of which exacerbate its prevalence with advancing age (Krijthe, B.P. et al., 2013; Schnabel, R.B. et al., 2015). AF is associated with severe complications, including thromboembolism, myocardial infarction, stroke, and the worsening of heart failure (Staerk et al., 2017; Catanese and Hart, 2019). Despite its prevalence, the underlying mechanisms of AF are not completely understood.

In AF, the atrial myocardium becomes hyperexcitable with a shortened refractory period, often triggered by ectopic atrial activity and reentry pathways. These mechanisms contribute to both electrical and structural remodeling (Iwasaki et al., 2011; Allessie et al., 2002), with angiotensin II (Ang II) playing a crucial role.

Ang II, a central component of the renin-angiotensin system, is frequently elevated in pathological conditions. It stimulates the production of reactive oxygen species, and promotes inflammation, fibrosis, and apoptosis through multiple pathways mediated by the AT1 receptor, a G-protein-coupled receptor. These processes result in significant electrical and structural alterations in the atria, thereby exacerbating fibrosis and accelerating the progression of AF (Forrester et al., 2018).

Extensive research indicates that Ang II is crucial in initiating key inflammatory processes. It increases vascular permeability, initiates inflammation, recruits inflammatory cells, and activates immune responses through chemotaxis and differentiation (Yusuke et al., 2003). Further studies suggest that Ang II not only induces inflammation but also impacts ion channels and currents, contributing to the atrial electrical and structural remodeling seen in AF (Jia et al., 2012).

Inflammation plays a significant role in the pathogenesis of AF by promoting the accumulation of inflammatory mediators in the atrial tissue, impacting both its structural and electrical properties (Vyas et al., 2020). Elevated levels of inflammatory markers such as CRP, interleukins (ILs), TNF, TGF-β, NF-κB, and NLRP3 have been observed in patients with AF (Sinner et al., 2014; Brezinov et al., 2021; Gungor et al., 2013; Allah et al., 2019; Fu et al., 2015; Guo, 2019; Deng et al., 2011; Lu, 2024; Xu et al., 2022; Yao C. et al., 2018). These factors disrupt electrical conduction by altering calcium homeostasis and connexins, thus increasing susceptibility to AF. Additionally, inflammatory mediators influence signaling pathways that promote atrial fibrosis (Hu et al., 2015; Hoffmann et al., 2016).

The interplay between inflammation, Ang II, and AF is intricate. While the influence of inflammation on Ang II-mediated signaling in AF is recognized, it is not yet fully elucidated. This article systematically reviews recent research on the key inflammatory pathways influenced by Ang II in AF and examines the roles of various inflammatory factors. Targeting these inflammatory pathways may offer novel insights into contemporary anti-inflammatory molecular mechanisms.

## 2 AngII and atrial fibrillation

Ang II plays a pivotal role in atrial electrical remodeling by modulating ion channels and currents, thereby substantially contributing to the development of AF. Via the AT1R, AngII increases intracellular sodium levels and enhances sodium-calcium exchange. This activity stimulates calcium-dependent potassium and chloride channels, effectively shortening the atrial refractory period (Iwasaki et al., 2011). Furthermore, AngII facilitates the release of calcium from the sarcoplasmic reticulum, thereby shortening the duration of the action potential (Nakashima and Kumagai, 2007).

Ang II is implicated in the upregulation of T-type calcium currents and the inhibition of L-type channels, thereby shortening the plateau phase of the action potential, which promotes rapid atrial excitation and facilitates atrial remodeling (Tsai et al., 2007). It also impacts potassium currents by inhibiting Ito and Ikur, while amplifying Ik1. These changes degrade structural proteins and alter potassium channel expression (Gu et al., 2014; Brundel et al., 2002). Additionally, Ang II induces atrial insufficiency and aberrant Ca^2+^ handling in AF models through the activation of CaMKII, which modulates RyR2 and results in Ca^2+^ leakage (Aonuma et al., 2022). Activation of CaMKII also increases CREB phosphorylation, enhancing the transcription of KCNJ2 and CACNA1C. This upregulation impacts both potassium and calcium channels, further promoting AF (Li et al., 2024).

**FIGURE 1:**
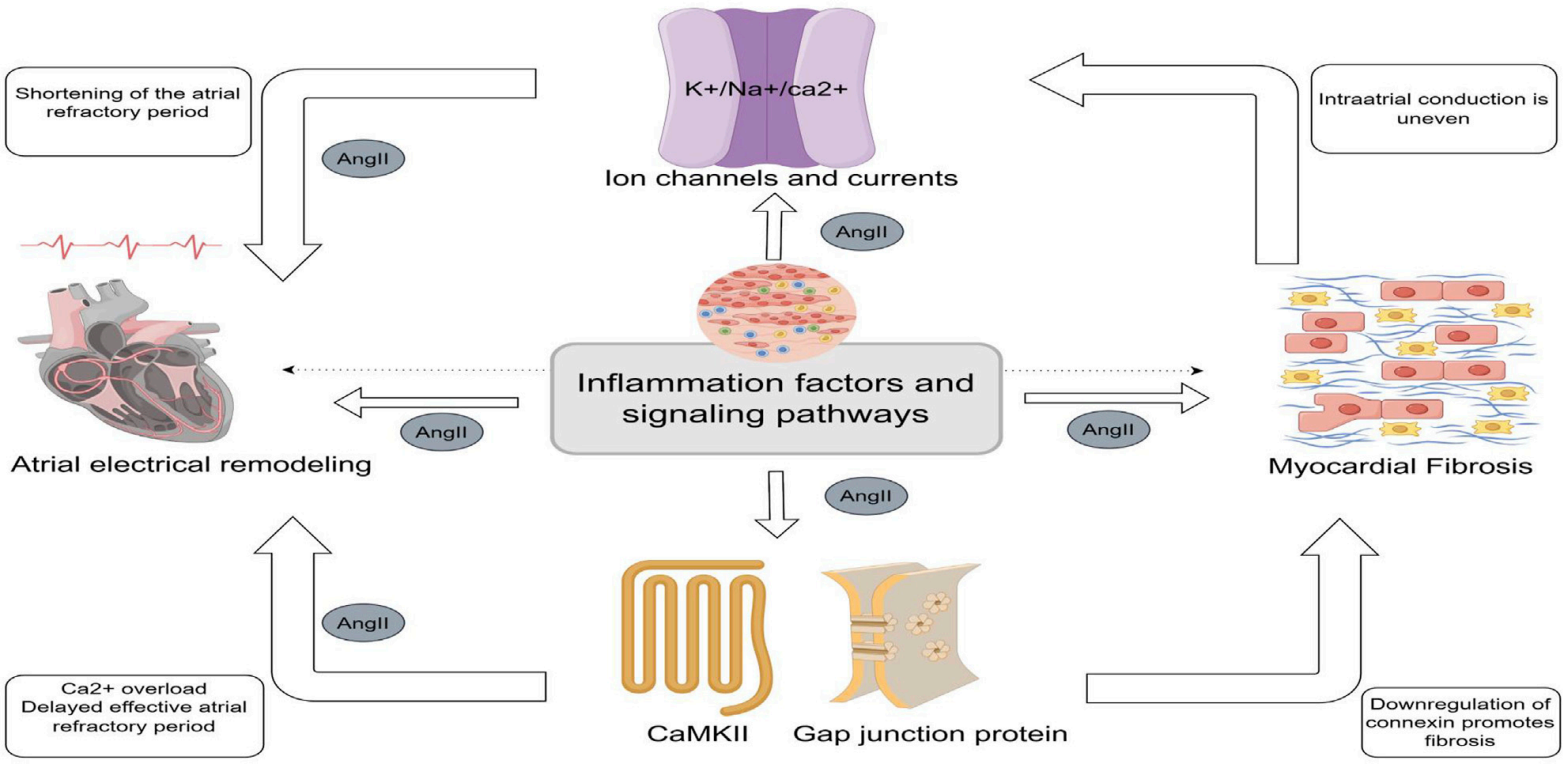
Central illustration (By Figdraw). Central role of inflammation in Angiotensin II- Induced Atrial Fibrillation. Inflammatory factors and inflammatory signaling pathways can contribute to atrial fibrillation by modulating ion channels and ion currents, calmodulin, and gap junction proteins to promote shortening and delaying of the effective atrial opriod. Myocardial fibrosis is the result of the co-regulation of Angiotensin II and multiple inflammatory signaling pathways. Fibrosis can lead to heterogeneous electrical signaling in the atria, affecting ion channels and currents and contributing to the occurrence of electrical remodeling.

As a well-documented pro-fibrotic agent, AngII’s interaction with the AT1R promotes myocardial fibrosis through multiple signaling pathways. This fibrosis contributes to intra- and inter-atrial conduction inhomogeneities, creating localized zones of refractoriness and a substrate conducive to the progression of AF (Li et al., 1999). This process is closely linked to inflammation (Jia et al., 2012). Moreover, higher levels of AngII correlate with more severe atrial fibrosis and a higher incidence of AF (Jansen et al., 2019). Ang II and inflammation drive atrial electrical remodeling and fibrosis by modulating ion channel function, thereby exacerbating atrial structural and electrical abnormalities (Figure 1).

## 3 Inflammation and electrical remodelling

Atrial electrical remodeling encompasses alterations in ion channel remodeling, ion current properties, and gap junction proteins, with inflammation playing a significant role in these processes (Ma et al., 2018). During experiments involving rapid atrial pacing in dogs, there is an observed increase in the expression of KCa3.1, a channel that regulates the repolarization phase of the cardiac action potential. This upregulation is mediated by the inflammation-related PI3K/AKT pathway (Yuntao et al., 2024). Inhibition of KCa3.1 has been shown to reduce macrophage polarization and prevent AF during sustained rapid pacing (He et al., 2021).

Furthermore, NF-κB activation induces its translocation to the nucleus, where it regulates the transcription of the KCa3.1 gene. Inhibiting NF-κB can attenuate the associated inflammation (Chen H et al., 2024). The interaction between RyR2 and CaMKII influences calcium transients and sarcoplasmic reticulum (SR) Ca^2+^ release, while the NLRP3/IL-1β pathway activates Ca2^+^/CaMKII, inducing Ca^2+^ release and arrhythmia. Increased CaMKII activity has been found to inhibit PI3K/AKT signaling, elevating the risk of AF (Shuai et al., 2023; Jiang et al., 2019).

Gap junction proteins such as Cx40 and Cx43, which are essential for cardiomyocyte coupling, are downregulated in inflammatory states. This downregulation alters atrial structure and conduction, promoting AF (Sawaya et al., 2007; Friedrichs et al., 2011). CX43 hemichannels mediate peripheral inflammatory signals, triggering the release of pro-inflammatory factors such as IL-1β and TNF-α (Ahmad et al., 2012). AngII may inhibit Cx43 by decreasing AMPK phosphorylation *via* K_ATP_ channels and activates p38, which overphosphorylates Cx43, disrupting cellular coupling and electrical remodeling (Wang, 2023). The inhibition of Cx43 attenuates JNK signaling and reduces the release of inflammatory mediators (Tien et al., 2021). Additionally, Cx43 hemichannels are involved in NLRP3 vesicle assembly and activation; the Cx43 inhibitor Gap26 has been shown to reduce inflammation markers (Wang WB et al., 2022). The involvement of ion channels and gap junction proteins in atrial electrical remodeling and fibrosis is illustrated in Figure 1.

## 4 Inflammation and fibrosis

**FIGURE 2:**
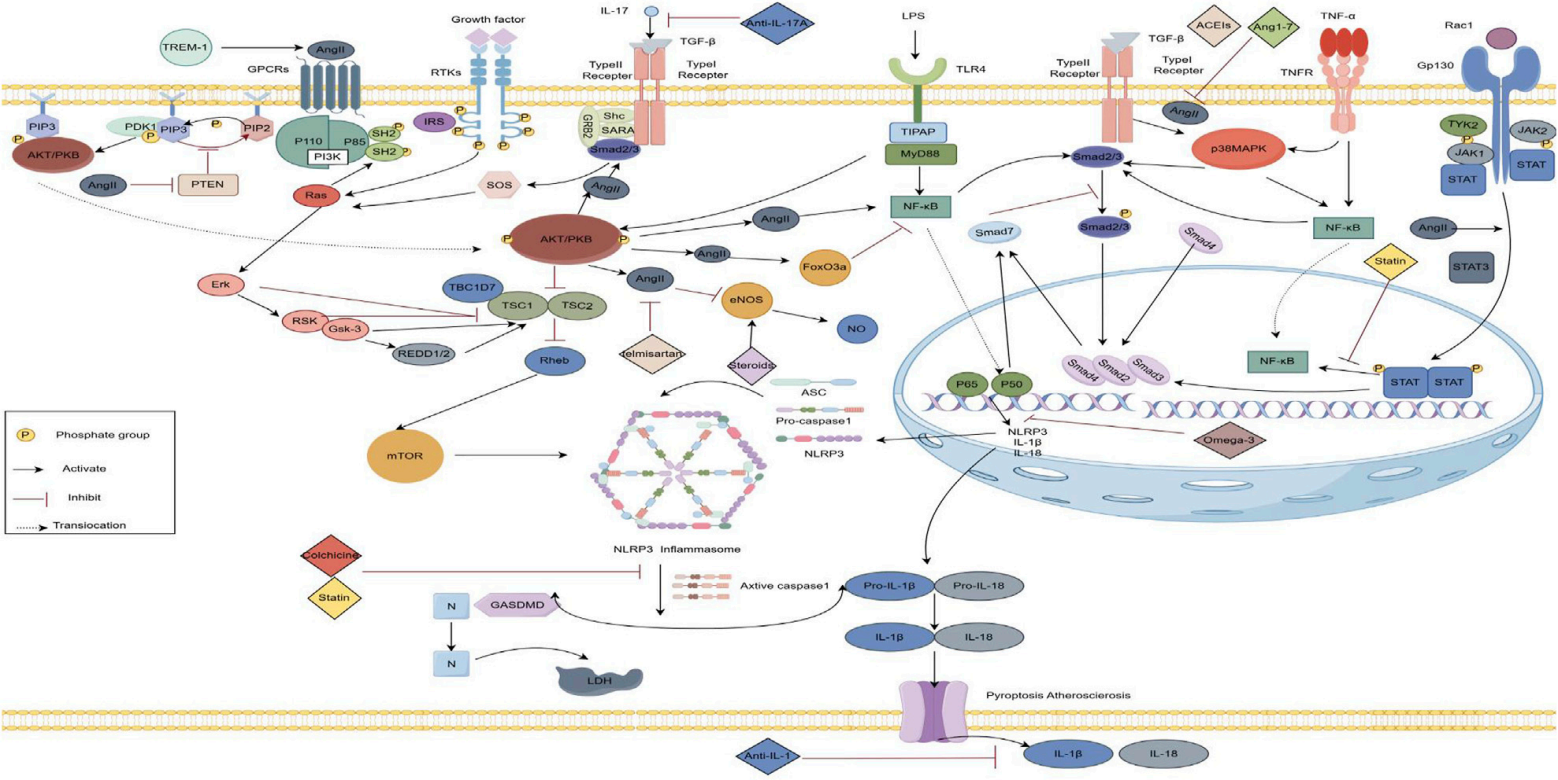
Inflammatory crosstalk and anti-inflammatory mechanisms in the angiotensin II-induced fibrosis pathway. (By Figdraw) AngII mediates the PI3K/AKT signaling pathway and participates in upstream PTEN, downstream TGF-ß, m TOR, FoxO3a and e NOS signaling pathways, as well as the P38MAPK, JAK/STAT3 and NF-κB signaling pathways, and NF-κB further activates the NLRP3 inflammatory vesicles, releasing IL-1ẞ, IL-18.Anti-inflammatory drugs act on the corresponding inflammatory pathways. TLR, Toll-like Receptor; IGF 1, Insulin-like growth factor 1 ;TNF, Tumor necrosis factor; TGF-B, Transforming growth factor-ß; IL, Interleukins; PI3K,Phosphatidylinositol 3-kinase;PIP3,Phosphatidylinositol Trisphosphate;PDK,Phosphatidylinositol-dependent kinase;FoxO3a, Forkhead box 03;mTOR, The Mechanistic Target of Rapamycin;e NOS,Endothelial nitric oxide synthas;JAK,Janus tyrosine Kinase;STAT,Signal Transducer and Activator of Transcription; NF-κB, Nuclear factor kappa-B protein;NLRP3, NOD-, LRR-, and pyrin domain-containing protein 3.

Atrial myocardial fibrosis, a defining feature of structural remodeling in AF, arises from a complex interplay of pro-fibrotic signaling pathways, inflammation, and oxidative stress (Tan and Zimetbaum, 2011). AngII plays a central role in these processes by engaging G-protein-coupled receptors to initiate various signaling cascades involved in intracellular, nuclear, and extracellular inflammatory responses. Figure 2 illustrates the interplay of inflammatory pathways.

### 4.1 TGF-β signaling pathway

The TGF-β signaling pathway, critical for regulating cellular proliferation, differentiation, immune responses, extracellular matrix synthesis, and inflammation, acts as a potent chemokine for cardiac fibroblasts. It promotes myocardial fibrosis by facilitating collagen deposition and suppressing its degradation (Xiao and Zhang, 2008). IL-17 enhances AngII-induced proliferation of atrial fibroblasts and fibrosis by upregulating TGF-β1 (Zhang, 2017). The TGF-β1/Smad pathway, integral to myocardial fibrosis, is regulated by the activation of AngII, NF-κB, and PI3K pathways (Hirsh et al., 2015). TGF-β1 synthesized by fibroblasts binds to TβRI/TβRII receptors, initiating a cascade that transmits signals to the nucleus *via* Smad proteins. Paced rabbit heart tissues show elevated levels of AngII, TGF-β1, and phosphorylated Smad2/3, alongside reduced Smad7, a critical negative regulator (He et al., 2011). Melatonin inhibits AngII-mediated TGF-β/Smad signaling, influencing atrial remodeling and AF (Xie et al., 2022). Additionally, GPR30 mitigates AngII-induced fibrosis by upregulating Smad7 expression (Liu et al., 2022).

### 4.2 PI3K/AKT signaling pathway

This pathway is central to inflammatory responses, linked to mediators like NLRP3, interleukins, and TNF-α (Guo et al., 2012). Dog studies with AF show reduced mRNA levels of PI3K/AKT in atrial tissues, alongside increases in TNF-α, IL-6, XO, and ROS in peripheral blood (Stark et al., 2015). Both mRNA and protein expressions of PI3K and p-AKT are downregulated in myocardial tissues of rats, where inflammatory factors are notably elevated, exacerbating myocardial fibrosis (Kang, 2019). AngII activates PI3K/AKT *via* GPCRs, promoting myocardial hypertrophy and fibrosis. LY294002, a PI3K/AKT inhibitor, reduces AngII-induced inflammation and myocardial fibrosis by lowering IL-6 and TNF-α levels (Zhu et al., 2024).

PTEN, an upstream regulator of PI3K-AKT, when degraded, enhances cardiac hypertrophy and myocardial fibrosis (Cao et al., 2019). Liraglutide moderates AngII-induced proliferation of cardiac fibroblasts and extracellular matrix deposition *via* the miR-21/PTEN/PI3K pathway (Wang et al., 2023). The immunoproteasome subunit PSMB10 alleviates myocardial fibrosis by reducing PTEN degradation and suppressing AKT1 activation (Li et al., 2018). The mTOR pathway downstream of PI3K/AKT contributes to fibrosis by promoting collagen production and myofibroblast transformation (Wu et al., 2021; Luo, 2023). Rapamycin, by inhibiting mTOR, diminishes inflammation and reverses cardiac remodeling (Liang, 2023). The PI3K/AKT/mTOR pathway also activates NLRP3 inflammasomes, releasing pro-inflammatory cytokines like IL-1β, IL-6, and IL-18 (Tai et al., 2022). FoxO3a, targeted by AKT, is upregulated in AngII-induced cardiac fibroblasts, fostering labile AF through enhanced fibrosis (Lin et al., 2024). FoxO3a knockdown markedly reduces fibroblast proliferation, migration, and collagen secretion (Lin et al., 2024). IGF-1R promotes myocardial fibrosis through activation of the PI3K/Akt/FoxO3a signaling pathway and predisposes to AF development and maintenance (Zhang, 2024). TREM-1 activation enhances susceptibility to AF by modulating the PI3K/AKT/FoxO3a signalling pathway to mediate inflammation production and release (Chen X et al., 2024). In diabetic rats, atrial fibrosis correlates with the inactivation of the PI3K/AKT/eNOS axis (Chu, 2015), while H2S modulates this pathway to alleviate fibrosis (Xue et al., 2020). Furthermore, the PI3K/AKT pathway triggers the TGF-β/Smad pathway, contributing to myocardial fibrosis.

### 4.3 MAPK signaling pathway

MAPKs, serine/threonine kinases, are pivotal in cellular processes like proliferation, differentiation, and apoptosis. The MAPK signaling cascade, consisting of MAPK, MAPKK, and MAPKKK, is activated by AngII binding to AT1R, stimulating fibroblast proliferation and inducing cellular hypertrophy and apoptosis. Elevated AngII levels, *via* TNF-α, prompt ROS release, activating the ASK1/MEKK3/6 pathway, which in turn activates p38MAPK, ERK, and JNK pathways, increasing protein synthesis and cardiomyocyte hypertrophy (Yu et al., 2013; Zhang et al., 2019). IL-17A activates the p38MAPK and ERK1/2 pathways, enhancing fibroblast proliferation and migration (Valente et al., 2012). p38MAPK is crucial in activating inflammatory pathways, regulating cytokine expression and increasing MMP1 mRNA levels, exacerbating myocardial fibrosis (Reunanen et al., 2002). Phosphorylated p38 protein expression in atrial tissues correlates with increased myofibroblast numbers (Shintaro et al., 2020). Laccase ameliorates left atrial dilatation, inflammation, and fibrosis by modulating the p38MAPK/Smad3 pathway (Liu et al., 2019). The antifibrotic drug c-Ski reduces p38MAPK phosphorylation and exerts antifibrotic effects (Han et al., 2021).

### 4.4 Rac1/JAK/STAT3 signaling pathway

Rac1, a small GTP-binding protein of the Rho GTPase superfamily, induces myocardial ROS production through NADPH oxidase activation, increasing oxidative stress, inflammation, and collagen accumulation, which facilitates the development of AF (Adam et al., 2007). Rac1 participates in the Ang II-mediated JAK/STAT3 signaling pathway, upregulating the expression of type I procollagen α1 (COL1A1) and contributing to myocardial hypertrophy and fibrosis (Hattori et al., 2006). Furthermore, AngII activates Rac1, which upregulates connective tissue growth factor (CTGF), N-cadherin, and Cx43 (Adam et al., 2010). CTGF is pivotal in extracellular matrix remodeling and tissue fibrosis (Perbal, 2004). The increased expression and redistribution of Cx43 and N-cadherin may impair atrial electrical conduction and promote interstitial fibrosis (Rucker-Martin et al., 2006). Rac1 also activates the Rac1/ASK1/NF-κB pathway, inducing cardiomyocyte hypertrophy and structural changes in the heart (Hirotani et al., 2002).

STATs, nuclear transcription factors, are integral to the JAK/STAT pathway and are closely associated with pathways like MAPK, NF-κB, TGF-β/Smad, and integrin/ERK. In atrial myocytes, AngII may activate STAT3 *via* Rac1 or a JAK/TYK-independent mechanism. In atrial fibroblasts, AngII-induced STAT1 activation requires Rac3-mediated autocrine or paracrine signaling (Ausma et al., 2008; Tsai et al., 2008). Rac1 membrane translocation and STAT3 activation drive structural remodeling and inflammatory responses in pacing-induced persistent AF. AngII also activates STAT3 by binding to the AT1 receptor, promoting the expression of matrix metalloproteinase 1 (MMP-1) and MMP-2 in atrial fibroblasts (Zheng et al., 2014). STAT3 further promotes Smad2/3 expression, contributing to fibrosis (Li, 2019). Inflammatory factors like TNF-α, IL-6, and IL-4 activate and regulate the JAK-STAT pathway, influencing cardiac hypertrophy and myocardial remodeling (Butler et al., 2006; Dawn et al., 2004; Fischer and Hilfiker-Kleiner, 2008). Additionally, anti-inflammatory factors IL-10 and IL-11 mitigate fibrosis, prevent apoptosis, and reduce inflammatory responses by activating STAT3 (Krishnamurthy et al., 2009; Obana et al., 2010).

### 4.5 NF-κB/NLRP3 signaling pathway

NF-κB, a key intracellular transcription factor, is activated by pro-inflammatory stimuli like TNF-α, IL-1, and bacterial products such as LPS. It induces the expression of genes encoding various cytokines (e.g., IL-1, IL-2, IL-6, IL-12, IFN-β, TNF-α, G-CSF, GM-CSF), enhancing the inflammatory response (Hu et al., 2018). NF-κB and TNF-α protein expression, along with inflammatory cell infiltration, were significantly elevated in the atrial tissues of patients with atrial fibrillation and in rats (Fan and Xue, 2012). Activated NF-κB induces myocardial fibrosis by promoting PICP production, modulating collagen tension, and reducing type I collagen accumulation (Fan, 2014). TNF-α also activates the NF-κB signaling pathway, promoting lung fibrosis in mice (Di et al., 2009). Toll-like receptors (TLRs) bind to MyD88, activating NF-κB, which triggers transcription and synthesis of pro-inflammatory cytokines like TNF-α, IL-1β, and IL-6, contributing to the immune-inflammatory response (Lee et al., 2016). Clinical studies show higher expression levels of NF-κB, TLR4, and MyD88-related molecules in AF patients compared to healthy individuals (Xu et al., 2018). High-fat diets increase susceptibility to ventricular arrhythmias by activating the TLR4/MyD88/CaMKII/NF-κB pathway, resulting in left ventricular hypertrophy, fibrosis, and reduced ion channel protein expression (Shuai et al., 2019). TLR4 antagonists prevent myocardial fibrosis by inhibiting the TLR4/MyD88 pathway (Mian et al., 2019).

NLRP3, a downstream component of NF-κB, is a multiprotein complex composed of NLRP3, pro-caspase-1, and ASC, abundantly expressed in cardiac fibroblasts (Guan et al., 2022). Recognition of stressors like TRAF6 and TLR-mediated activation of NF-κB upregulates inflammatory vesicle-associated proteins, including IL-1β and IL-18. This process involves monomer assembly into a complex and the conversion of pro-IL-1β and pro-IL-18 into their active forms, mediating inflammatory responses (Celias et al., 2019). The NLRP3/IL-1β pathway induces inflammation through ROS production, with ROS acting as secondary messengers to promote further inflammation (Checa and Aran, 2020). The activation of NLRP3 inflammasomes in atrial cardiomyocytes initiates a fibroinflammatory cascade involving cardiomyocytes, immune cells, and fibroblasts, thereby driving atrial fibrosis, remodeling, and the progression of AF (Dzeshka et al., 2015). These vesicles promote cytokine and collagen production by myofibroblasts, contributing to structural remodeling, RyR2 channel remodeling, atrial enlargement, stromal fibrosis, and abnormal gap junction protein distribution *via* RYR2 upregulation (Li and Brundel, 2020; Li et al., 2020). These abnormalities occur through the NF-κB/NLRP3 pathway. Elevated NLRP3 and IL-1β levels have been observed in aged rat atria with AF, linked to increased TLR4/NF-κB/NLRP3 pathway activation. Blockade of TRPV4 prevents AF and reduces fibrosis in aseptic pericardial mice by inhibiting the ERK/NF-κB/NLRP3 pathway (Yang et al., 2022; Zhang H et al., 2022). Furthermore, the NLRP3/IL-1β/MyD88 pathway triggers the TGF-β/Smad pathway, promoting fibrosis development (Alyaseer et al., 2020).

## 5 Anti-inflammatory treatment of atrial fibrillation

### 5.1 RAAS inhibitors

Ang II promotes AF and myocardial fibrosis *via* diverse inflammatory signaling pathways. Inhibiting the renin-angiotensin-aldosterone system (RAAS) can reduce inflammation and reverse atrial electrical and structural remodeling (Roşianu et al., 2013). Meta-analyses have demonstrated that angiotensin-converting enzyme inhibitors (ACEIs) and angiotensin receptor blockers (ARBs) decrease the risk of AF in patients with hypertension, heart failure, myocardial infarction, and hypertrophy. These agents are effective in treating paroxysmal and persistent AF and in preventing recurrence post-electrical cardioversion or catheter ablation (Jibrini et al., 2008; Li et al., 2013; Huang et al., 2018; Zhao et al., 2020). ACEIs reduce AF duration by decreasing frequency-dependent APD90 in the atrial myocardium and lowering MAPK and AngII levels (Nakashima et al., 2000; Liu et al., 2011). Chlorosartan decreases AF susceptibility by modulating ion channel expression, particularly increasing Ito (Kv4.2) and decreasing Kv1.5 and Kir2.1/2.3 (Saygili et al., 2007). Furthermore, telmisartan reduces AF in hypertensive rats by enhancing PI3K/AKT/eNOS signaling (Wang, 2015). Ang1-7, an endogenous antagonist of AngII, improves ventricular remodeling by interfering with p38MAPK and reducing inflammatory mediators such as TGF-β and TNF-α (Bai, 2021).

However, aldosterone antagonists like spironolactone and eplerenone also play a role in delaying the recurrence of paroxysmal and post-catheter ablation AF (Dabrowski et al., 2010; Ito et al., 2013). Eplerenone, in particular, reduces atrial dilation and fibrosis without affecting electrical remodeling (Takemoto et al., 2017). Despite these benefits, a retrospective study indicated that ACEIs and ARBs might increase AF risk post-cardiac bypass grafting, highlighting the complexity of the RAAS-AF relationship (Miceli et al., 2009). This is related to the limitations of clinical detection tools, as it is difficult to detect all clinical AFs before the development of atrial remodelling. There are no reports that inhibition of RAAS can inhibit and reverse atrial remodelling, which may explain the clinical inconsistency. In addition ARNI as a treatment for heart failure, which contains ARBs, has shown potential to reverse remodelling (Abboud and Januzzi, 2021),but has not been used in AF, and future focus should be on the role of RAAS inhibitors/ARNIs on reverse AF remodelling.

### 5.2 Colchicine

Colchicine, a microtubule inhibitor, downregulates various inflammatory pathways and modulates innate immunity by inhibiting protein polymerization and microtubule formation (Lawler et al., 2021). It reduces the activity of NLRP3 inflammasomes and decreases the secretion of pro-inflammatory cytokines and interleukins, such as IL-1β and IL-6 (Amaral et al., 2023). Colchicine has shown efficacy in animal models by reducing atrial fibrosis driven by inflammatory responses, impacting pathways involving IL-1β/IL-6, p38, AKT, and STAT3, as well as NLRP3 inflammasome assembly and activation (Liu, 2019; Misawa et al., 2013).

Although promising in animal studies, clinical trials have produced mixed outcomes. Some studies report a prophylactic effect against postoperative AF, while others show increased risks of side effects such as gastrointestinal issues, which may influence electrolyte balances and increase AF susceptibility (Wang X. et al., 2022; Deftereos et al., 2012; Gudbjartsson et al., 2020; Duarte, 2014; Conen et al., 2023). Conflicting findings also exist regarding the prevention of pericarditis associated with postoperative AF (Ahmed et al., 2023; Mohanty et al., 2023). The variability in outcomes may result from differences in study designs, dosages, timing, duration, and drug combinations used (Attia and Hiram, 2024).

### 5.3 Steroids

Steroid therapy, known for its anti-inflammatory effects, shows mixed efficacy in preventing AF (Larsson et al., 2021). While some studies indicate that steroids like prednisolone can reduce electrophysiological changes and fibrosis markers associated with atrial tachycardia remodeling (Shiroshita-Takeshita et al., 2006; Zhang Y. et al., 2022), others have reported potential adverse effects, including the induction of atrial arrhythmias (Iwasaki et al., 2022). Steroids have been effective in preventing AF recurrence post-catheter ablation (Kim et al., 2015) and reducing inflammatory markers (Iskandar et al., 2017). However, they have not consistently improved clinical outcomes post-AF ablation. Dosage plays a critical role, with some studies suggesting that moderate doses may reduce the risk of postoperative atrial fibrillation (POAF) more effectively than either high or low doses (Viviano et al., 2014; Liu et al., 2014; Chai et al., 2022; Ho and Tan, 2009).

### 5.4 Statin therapy

Statins are recognized for their anti-inflammatory properties and their ability to modulate multiple pathways that could reduce AF incidence (Oraii et al., 2021). They have been shown to inhibit NLRP3 inflammasome activation and modulate the PDGF/Rac1/NF-κB signaling pathway, thereby reducing atrial fibrosis (Soucek et al., 2015; Peña et al., 2012). While statins have been effective in reducing POAF in various surgical contexts, they have not significantly changed the incidence and prognosis of POAF in all studies (Kuhn et al., 2021; An et al., 2017; Yan et al., 2014; Allah et al., 2019; Bonano et al., 2021; Fiedler et al., 2021; Oliveri et al., 2022; Yu et al., 2022). However, they have been shown to reduce the risk of heart failure, stroke, and all-cause mortality, with stronger statins demonstrating greater efficacy (Choi et al., 2024; Huang et al., 2023; Pastori et al., 2021; Zhang et al., 2020). Low-dose statin pretreatment has also been found to improve initial stroke severity and functional outcomes at 90 days (Dong et al., 2019). Despite these benefits, current guidelines do not recommend statins specifically for AF management due to study heterogeneity.

### 5.5 Polyunsaturated fatty acids (PUFAs)

Omega-3 fatty acids, a type of polyunsaturated fatty acid found in fish oil, are known for their antioxidant properties. They regulate ion channels, mitigate rapid-pacing-induced shortening of atrial refractory periods, and regulate myocardial CX40 expression, ensuring normal electrical signaling. They also inhibit NF-κB-mediated NLRP3 inflammasome activation, reducing inflammation and myocardial fibrosis (Szeiffova et al., 2020). While animal studies suggest that PUFA treatment reduces the risk of AF and atrial structural remodeling (Ninio et al., 2005; Sakabe et al., 2007), clinical trials have shown mixed results. Some trials suggest a beneficial effect following coronary artery bypass grafting (Calò et al., 2005), but most report no effect or even exacerbation of AF development, postoperative AF, or post-recovery AF (Saravanan et al., 2010; Albert et al., 2021; Kowey et al., 2010; Cao et al., 2012; Kalstad et al., 2021; Lombardi et al., 2021; Qi et al., 2023). The risk of atrial fibrillation increases with higher doses (Gencer et al., 2021). Variations in patient profiles, surgeries, arrhythmia definitions, and monitoring methods may account for these conflicting findings. Overall, current evidence does not support the use of omega-3 fatty acids for AF treatment.

### 5.6 Antibody-targeted therapy

Although anti-inflammatory therapy for AF has been proposed for over 2 decades, its targeted therapeutic strategies remain under development. The CANTOS trial demonstrated that the monoclonal anti-IL-1β antibody canakinumab reduced major cardiac events and heart failure hospitalizations in CAD patients with elevated hsCRP (Ridker et al., 2011). IL-6 plays a role in AF pathogenesis by promoting neutrophil-induced atrial fibrosis and abnormal calcium currents. IL-6 antibody treatment attenuates atrial fibrosis and reduces AF risk in aseptic pericarditis rat models (Liao et al., 2021). IL-10, an anti-inflammatory cytokine, has been shown to mitigate high-fat diet-induced AF, with IL-10 knockout mice exhibiting more severe fibrosis and AF progression, both alleviated by IL-10 administration (Kondo et al., 2018). IL-17A, a pro-inflammatory cytokine from Th17 cells, activates NF-κB and MAPK pathways, contributing to fibrosis and AF in AngII-induced models (Liu et al., 2012). Anti-IL-17A monoclonal antibodies have reduced AF in transesophageal atrial pacing models (Fu et al., 2015). Additionally, IL-33 mediates atrial remodeling by activating CaMKII/RyR2 and NF-κB/NLRP3 signaling pathways, promoting arrhythmogenesis. Anti-ST2 antibodies have reduced IL-33-mediated atrial fibrosis and arrhythmias in mice (Tzu et al., 2024). These findings indicate that the targeted blockade of inflammatory factors and associated pathways holds significant potential, although further clinical trials are required to validate their efficacy.

## 6 Conclusion

AF is one of the most prevalent cardiac arrhythmias, significantly influenced by the RAAS. AngII, a crucial component of this system, facilitates atrial electrical remodeling by modulating ion channels and currents, thereby contributing to the development of AF. Beyond its role in electrical remodeling, AngII also serves as a potent pro-fibrotic agent, driving atrial fibrosis through various inflammatory signaling pathways, including TGF-β, PI3K/AKT, MAPK, Rac1/JAK/STAT3, and NF-κB/NLRP3. This mediation of inflammation and the resultant crosstalk among these pathways are instrumental in the progression of AF. Moreover, AF itself can intensify the inflammatory response, thus perpetuating a vicious cycle that accelerates disease progression.

Recent research underscores the pivotal role of inflammation in the initiation and perpetuation of AF. Inflammation fosters a pro-arrhythmic environment, disrupts the electrophysiological properties of atrial myocytes, and promotes structural remodeling. Targeting inflammation has emerged as a promising strategy for the prevention and treatment of AF. Further investigation into the molecular mechanisms underlying Ang II-induced inflammation is essential to identify novel therapeutic targets. A variety of anti-inflammatory therapies, including RAAS inhibitors, colchicine, steroids, statins, and polyunsaturated fatty acids, have shown potential in reducing AF incidence and improving clinical outcomes, although the results remain inconsistent. The role of ARNI and RAAS inhibitors in reversing cardiac remodeling warrants further investigation. Additionally, the potential of combination therapies integrating anti-inflammatory agents with existing AF treatments should be explored. Large-scale randomized controlled trials are needed to evaluate the efficacy of anti-inflammatory strategies in AF management and to develop early interventions aimed at preventing the onset and recurrence of AF. Despite these challenges, the central role of inflammation in arrhythmogenesis highlights a potential path for innovative treatment approaches.
