# Supplementary material for: Inflammation pathways as therapeutic targets in angiotensin II induced atrial fibrillation
Source: Front Pharmacol. 2025 Mar 3;16:1515864. doi: 10.3389/fphar.2025.1515864 (PMC11911380; doi:10.3389/fphar.2025.1515864)
Supplement: Supplementary file 1 [file Supplementaryfile1.pdf]

Supplementary Material

1 Supplementary Tables

Table 1 Abbreviations Glossary

ID	Abbreviations	Full Name
1	AF	Atrial fibrillation
2	CRP	C-reactive protein
3	ILs	Interleukins
4	TNF	Tumor necrosis factor
5	TGF-β	Transforming growth factor-β
6	NF-κB	Nuclear factor kappa-B protein
7	NLRP3	NOD-, LRR-, and pyrin domain-containing protein 3
8	PI3K	Phosphatidylinositol 3-kinase
9	PIP3	Phosphatidylinositol Trisphosphate
10	PDK	Phosphatidylinositol-dependent kinase
11	TLR	Toll-like Receptor
12	LPS	Lipopolysaccharide
13	mTOR	The Mechanistic Target of Rapamycin
14	MAPKK	MAPK Kinase

15	MAPKKK	The Kinase of the MAPK Kinase
16	AERP	AF Effective Refractory Period
17	IACT	Interatrial Conduction time
18	MMP	Matrix Metalloproteinase
19	Rac1	Atrial fibroblasts
20	USP38	Ubiquitin-specific Protease 38
21	ASC	caspase recruitment domain
22	TRPV4	Transient Receptor Potential Vanilloid 4
23	ER	Endoplasmic Reticulum
24	Th17	Helper T cells 17
25	PRR	Pattern Recognition Receptors
26	NLR	Neutrophil/Lymphocyte Ratio
27	LAEF	LA Emptying Fraction
28	POAF	Postoperative AF
29	CTGF	Connective Tissue Growth Factor
30	COLA-I	collagen-I
31	COLA-Ш	collagen-Ш

Table 2 Inflammatory factors associated with atrial fibrillation

Inflam-matory factors	Source	Presence site	Role in atrial fibrillation
CRP	Wide range of inflammatory markers	Serum	Elevated CRP levels correlate with the occurrence and recurrence of atrial fibrillation and postoperative atrial fibrillation episodes(Sinner et al., 2014).CRP activates the NF-κB, TGF-β, and Smad pathways to interfere with cell viability, and induces an inflammatory response through Toll-like receptor 4 (TLR4), which mediates the onset of atrial fibrillation and concomitant atrial fibrosis (Perelshtein et al.,2021;Sun et al,2019).
Il-1β	Pro-inflammatory cytokines secreted by monocytes and macrophages	Serum	IL-1β is associated with the progression of paroxysmal AF to persistent AF(Gungor et al. ,2013). IL-1β activates the downstream protein kinase PKCε pathway and increases the susceptibility to AF(Loeser et al.,2008), and also regulates the oxidation and phosphorylation of intracellular calmodulin-dependent proteinkinase II (CaMKII), which increases spontaneous contraction frequency and induces the development of AF in cardiomyocytes(Monnerat et al .,2021). frequency of cardiomyocytes, inducing atrial fibrillation.
IL-6	Multipotent cytokines produced by various cells such as macrophages and fibroblasts	Serum、Atrial tissue	IL-6 can act as an independent risk factor for non-valvular AF and also has a positive correlation with postoperative AF(Sharma et al.,2018).IL-6 promotes local inflammatory responses by regulating the differentiation of CD4+ T cells and B cells(Zhou et al.,2007), and participates in the P38, c-Jun amino-terminal kinase (JNK) and NF-κB inflammatory response pathways, causing AF(Dobrev et al.,2019).
IL-10	an anti-inflammatory cytokine secreted mainly by	Serum、Atrial tissue	IL-10 levels correlate with type of AF and are higher in patients with persistent AF than in those with paroxysmal AF.IL-10 is associated with high-fat-induced inflammation and is involved in the NF-κB inflammatory signalling pathway, which

	monocytes		causes atrial inflammation, steatosis, fibrosis and atrial fibrillation(Kondo et al.,2018).
IL-17	An immune system pro-inflammatory cytokine produced by a subpopulation of Th17 cells	Serum、Atrial tissue	IL-17 correlates with recurrence of atrial fibrillation and type of atrial fibrillation after catheter ablation and is involved in NF-κB and mitogen-activated protein kinase signalling pathways to mediate the inflammatory response causing atrial fibrillation(Xu et al.,2019;Zhang et al.,2021;Miossec and Kolls.,2012).
IL-18	Pro-inflammatory cytokines produced by macrophages	Serum、Atrial tissue	IL-18 acts as an inflammatory marker of AF episodes and AF severity, and is positively correlated with LAD and is involved in atrial remodelling.IL-18 promotes the production of pro-inflammatory factors such as TNF-α, IL-1 and IL-6, which leads to intracellular calcium deposition in atrial myocytes, affecting cardiomyocyte degeneration, necrosis, apoptosis, and fibrosis, leading to the onset and persistence of AF(Guo et al.,2019).
TNF -α	An endogenous inflammatory mediator synthesised and secreted by various immune cells including macrophages and lymphocytes.	Serum、Atrial tissue	TNF-α correlates with AF severity and is positively correlated with left atrial internal diameter(Deng et al.,2011).TNF-α induces AF by regulating calcium channel proteins involved in either the p38 MAPK signalling pathway or the TGF-β/Smad2/3 signalling pathway that induces atrial fibrosis(Lakin et al.,2023;Liew et al.,2013).
TGF-β1	A pro-fibrotic cytokine	Serum、Atrial tissue	TGF-β1 levels contribute to the early diagnosis of atrial fibrillation and the prediction of recurrence risk, but are not associated with the progression of atrial fibrillation（Lu.,2024;Stanciu et al., 2018）.TGF-β1 regulates the expression of fibrosis-related genes, causing atrial fibrosis and increasing susceptibility to atrial fibrillation(Verheule et al.,2004).

NF-κB	A protein complex that is present in almost all animal cells	Serum、Atrial tissue	NF-κB correlates with the severity of atrial fibrillation and is positively associated with left atrial fibrosis(Xu et al.,2022).NF-κB regulates the transcription of inflammatory cytokines such as TNF, IL-1, IL-6, IL-8, IL-17, etc., and also inhibits the transcription of genes coding for cardiac Na channels, which induces electrical remodelling due to the abnormal expression of sodium channel proteins, and at the same time, NF-κB can activate the downstream NLRP3 and TNF-α, further inducing AF(Liu et al.,2013;Gao and Dudley.,2009; Chen et al.,2018).
NLRP3	Immune response mediators consisting of NLRP3, apoptosis associated speck like protein containing CARD (ASC) and extended apoptosis kinase 1 precursor (procaspase-1)	Serum、Atrial tissue	NLRP3 inflammatory vesicles are associated with episodes of AF and NLRP3 expression is increased in persistent AF compared to paroxysmal AF(Yao et al.,2018). Cardiomyocyte NLRP3 inflammatory vesicles contribute to aberrant release of calcium ions from the sarcoplasmic reticulum, leading to an increase in the inward sodium-calcium exchange current and a decrease in the outward current IK1, which induces spontaneous depolarisation and increases susceptibility to AF. In addition upregulation of myocyte enhancer factor 2c expression causes atrial hypertrophy and atrial fibrosis(Chen et al.,2018; Bai et al.,2018).

Table 3 AngII Antagonist Intervention in Atrial Fibrillation

Authors(Years)	Drug	Methods	Results
He X,et al. (2011)	AT(1) receptor antagonist losartan	Rapid atrial pacing (1000 ppm) was applied to the left atrium of rabbit hearts to induce atrial fibrillation and fibrosis. Changes in Ang II, TGF-β (1), phosphorylated Smad2/3 (P-Smad2/3), and Arkadia expression were assessed before and after the application of cloxartan.	Rapid atrial pacing resulted in a significant increase in the expression of Ang II, TGF-β (1), phosphorylated Smad2/3 (P-Smad2/3), Arkadia, and hydroxyproline synthesis. These changes were dose-dependently reversed by the AT(1) receptor antagonist cloxartan, suggesting involvement in AF-induced Ang II release and activation of AT(1) receptor-specific pathways.
Khatib R,et al.（2012）.	angiotensin-converting enzyme inhibitors/ angiotensin II-receptor blockers/aldosterone antagonists	Assessment of RAAS Inhibitors in New Onset Atrial Fibrillation by Searching Electronic Databases and Previous Systematic Evaluations After 1984	RAAS inhibition compared with conventional therapy or placebo reduced new onset atrial fibrillation ARBs showed a strong effect in the reduction of onset atrial fibrillation .
Wang, Q., et al.(2016）.	perindopril	Patients with paroxysmal AF who received radiofrequency ablation were randomized to a 3-month course of perindopril 8 mg once daily (perindopril group) or placebo (placebo group).	Perindopril is an effective and safe treatment for the prevention of AF recurrence after radiofrequency catheter ablation.
Sun, Y. L., et al.(2018).	Valsartan	Assessment of the Effect of Valsartan on Atrial Conduction Velocity and Connexins in Deafferented Atrial castrated Mice	Valsartan reduced the susceptibility of AF in castrated mice, which may be related to the inhibition of action potential prolongation and improvement of atrial conduction impairment.

Li, S. N., et al.(2022).	SAC/VAL	Eighteen AngII-induced SD rats were used and randomised to receive corn oil (CO), valsartan or sacubitril/valsartan for 24 days. Observation of AF episodes and myocardial fibrosis	Reduced atrial fibrosis, inhibition of atrial fibroblast proliferation, migration and differentiation, and reduced susceptibility to AF in the SAC/VAL group of rats, associated with p-Smad2/3, p-JNK and p-p38MAPK pathways
Huang, C. Y., et al .(2018).	ACEI/ARB	Retrospective study of 18 266 subjects with hypertrophic cardiomyopathy from January 1997 to December 201318 to assess the risk of new-onset atrial fibrillation in patients with hypertrophic cardiomyopathy from exposure to ACEIs or ARBs.	In patients with HCM, lower risk of new AF is observed in patients treated with either ACEIs or ARBs compared with those receiving neither of these medications.
Chaugai, S.,et al. (2016).	renin-angiotensin-aldosterone system (RAAS) blockers	A meta-analysis of 26 randomised controlled trials (RCTs) assessed the efficacy of RAAS blockers for the primary prevention of AF in patients with cardiovascular disease, including hypertension, myocardial infarction, and heart failure.	RAAS blockers are effective in suppressing AF in systolic heart failure.Angiotensin receptor blockers appeared to be slightly superior to angiotensin-converting enzyme inhibitors in primary and secondary prevention.
Jibrini, M. B., Molnar, J., & Arora, R. R. (2008).	ACEI/ARB	A meta-analysis of 11 randomised, controlled, parallel-design clinical trials assessing the effect of ACEIs or ARBs on the development of AF	inhibition of the RAAS reduced the RR of AF by 19% (RR 0.810, $P < 0.001$, 95% CI 0.759-0.865).
Li, T. J., et al.(2013).	renin-angiotensin-aldosterone system (RAAS) blockers	To assess the efficacy and safety of RAAS blockers in preventing AF recurrence based on 15 randomised controlled trials (RCTs) with a total of 3972 AF patients.	RAS inhibitors significantly reduced recurrent AF compared with non-RAS inhibitors (OR=50.95, 0% CI: 37.0-69.0, p<01.1) and showed beneficial effects in both paroxysmal patients and patients with persistent AF after cardioversion. However, administration of RAS inhibitors did not

			provide a greater survival advantage and lower incidence of adverse events compared to controls (OR=17.95,0% CI, 65.2-10.0, p=59.0; OR=94.95,0% CI: 65.1-35.0, p=73, respectively<>).
Dabrowski, R., et al.(2010).	spironolactone	A prospective randomised trial of 164 patients with recurrent AF assessed the rate of symptomatic AF episodes in four treatment groups (group A, spironolactone, enalapril and β-blocker; group B, spironolactone and β-blocker; group C, enalapril plus β-blocker; group D, β-blocker alone).	Spironolactone in combination with a β-blocker prevents AF episodes in patients with normal left ventricular function and a history of refractory paroxysmal AF
Ito, Y., et al. (2013).	eplerenone	To assess the effects of eplerenone and other conventional medications on clinical outcomes after RFCA in 161 patients with long-term persistent AF.	Eplerenone significantly improved the maintenance of sinus rhythm after RFCA in patients with long-standing persistent AF.
Li, D.et al,(2001).	enalapril	To assess the effect of enalapril on arrhythmogenic atrial remodelling and associated mitogen-activated protein kinase (MAPK) changes in a dog model of congestive heart failure (CHF).	Enalapril significantly reduced rapid beat-induced atrial angiotensin II concentrations and phosphorylation levels of c-Jun N-terminal kinase (JNK), extracellular signal-regulated kinase (ERK), and p38 kinase, and attenuated the effects of CHF on atrial conduction, atrial fibrosis, and mean AF duration.
Roşianu, Ş. H., et al.(2013).	ACEI/ARB	To study the effect of RAAS inhibitors on the recurrence of AF in 82 patients with paroxysmal atrial fibrillation and the correlation with inflammation.	ACE inhibitors and ARBs act on the cardiac matrix and reduce the inflammatory process, and may have a therapeutic-protective effect in reducing the burden of AF.
Boldt A , et	ACEI	Atrial tissue samples from patients with isolated	In patients undergoing ACE-I treatment: atrial structural

al. (2006).		chronic AF or sinus rhythm (SR) were selected to study the effect of ACEIs on collagen I, vascular endothelial growth factor (VEGF), and basic fibroblast growth factor (bFGF) protein expression.	remodeling was attenuated and the loss of atrial microcapillaries was prevented.
Zhao J, et al.(2020).	RAS inhibitors	A meta-analysis of 13 clinical studies involving 3661 patients with atrial fibrillation to assess the impact of RAAS inhibitors on AF recurrence rates after ablation	RAS inhibitors have shown significant benefit in reducing AF recurrence rates after catheter ablation.
Liu T,et al.(2016).	Mineralocorticoid receptor antagonists	A meta-analysis of five clinical trials was conducted to investigate the protective effects of saline corticosteroid receptor antagonists (MRAs) against AF.	Eplerenone significantly reduces the burden of AF ,MRA may be effective in AF prevention, especially in the HF setting.
Miceli A, et al. (2009).	angiotensin-converting enzyme inhibitor (ACEI)	This study evaluated the effect of pre-operative angiotensin-converting enzyme inhibitor (ACEI) therapy on early clinical outcomes after coronary artery bypass grafting (CABG).	Pre-operative therapy with ACEI is associated with an increased risk of mortality, use of inotropic support, PRD, and new onset of post-operative AF.
Wang W.W.(2015).	telmisartan	Assessment of the role of Ang II/AT1R and PI3K/Akt/eNOS signalling pathways in structural remodelling of hypertensive atria and the effect of telmisartan on susceptibility to atrial fibrillation in hypertensive rats	Timosartan simultaneously inhibited the AngII/AT1R signalling pathway and activated the PI3K/Akt/eNOS signalling pathway, restored the balance between the two signalling pathways, reversed the atrial structural remodelling, and reduced the susceptibility to atrial fibrillation in hypertensive rats.
Zhang FH,et al. (2019).	angiotensin-(1-7)	Selection of dogs with atrial fibrillation to assess the role of angiotensin-(1-7) on inflammatory factors and the P38MAPK signalling pathway in the	Ang-(1-7) has an anti-inflammatory effect in myocardial tissue, which is associated with inhibition of p38MAPK activation.

		course of atrial fibrillation	
Cao Y.(2021).	angiotensin-(1-7)	A mouse atrial myocyte (HL-1) inflammatory factor model of atrial fibrillation was established with the stimulating factor TNF-a (100ug/ml) to assess whether inflammatory factors leading to mouse atrial myocytes (HL-1) could act through the MKK/P38MAPKs pathway and whether it was protective of the pathway through ang1-7/MAS	The MKK-P38MAPKs pathway is involved in the inflammatory response of atrial myocytes (HL-1) in mice with atrial fibrillation, and ang1-7/MAS is protective of the pathway
Bai L. (2021).	angiotensin-(1-7)	In the present study, we investigated the role of Ang1-7 in the P38/MAPK pathway in HL-1 cells by TNF-α intervention in mouse atrial myocardial HL-1 cells	Ang1-7 can inhibit the phosphorylation of P38/MAPK, thus affecting the activation of the P38/MAPK pathway.Ang1-7 acts as a suppressor of inflammation by inhibiting the activation of P38 protein in HL-1 cells.
Nakashima, H., et al.(2000).	candesartan 、captopril	Selection of 24 dogs with atrial fibrillation to study the effects of candesartan and captopril on atrial electrical remodelling	In the candesartan and captopril groups, shortening of the AERP after rapid pacing was completely inhibited
Liu, E., et al.(2011).	Irbesartan 、Enalapril 、Ang-(1-7)	To investigate the effects of the ACE inhibitor enalapril, the angiotensin-receptor blocker (ARB) irbesartan, and Ang-(1-7) on the chronic atrial ionic remodeling.	APD90 changes were prevented by irbesartan and Ang-(1-7). Enalapril increased the density and gene expression of I(TO) compared with sham, Ang-(1-7) prevented the decrease of I(TO) and I(CaL) ($P < 0.05$ vs. control) and Kv4.3 mRNA expression.
Saygili, E., et al.(2007).	losartan	The study used a model of cultured atrial neonatal rat cardiomyocytes under conditions of stretch to provide insight into the mechanisms of the	Prevention of stretch-induced electrical remodeling might contribute to the clinical effects of losartan against AF.

		preventive effect of the angiotensin receptor-blocking agent losartan against AF on a molecular level.	
Takemoto, Y., et al.(2017).	eplerenone	Evaluation of the effects of eplerenone on atrial dilatation and electrical remodelling in atrial fibrillation by means of a randomised controlled study in 34 sheep with rapid atrial pacing	In the sheep model, EPL mitigates fibrosis and atrial dilation, modifies AF inducibility and AF complexity, and prolongs the transition to persistent AF in 26% of animals, but it does not prevent AF-induced electrical remodeling or AF persistence.
Yang SS,etal.(2008).	spironolactone	This study investigated the effects of spironolactone, a kind of aldosterone antagonist, on atrial electrical remodeling and fibrosis in CHF dogs induced by chronic rapid ventricular pacing.	Spironolactone treatment did not alter AERP duration, but this medicine dramatically decreased AERPd ($P < 0.05$), shortened intra- and inter-atrium conduction time ($P < 0.05$), and increased atrium CV. Moreover, spironolactone decreased the inducibility and duration of AF ($P < 0.05$), as well as atrial fibrosis ($P < 0.01$) induced by chronic rapid ventricular pacing.

Table 4 Study of anti-inflammatory treatment of atrial fibrillation

Authors (Years)	Targeted drug	Study population	Research contents	Results
Ocal, A. G., et al.(2020).	colchicine	FMF patients	The study evaluated the impact of 1 year of colchicine treatment on atrial and ventricular arrhythmogenic electrocardiographic (ECG) parameters in newly diagnosed FMF patients.	1-year long colchicine treatment in newly diagnosed FMF patients may decrease some of the ventricular repolarisation indices without negative changes in atrial arrhythmogenic ECG parameters

Wang, X., et al .(2022).	colchicine	Post-cardiac procedure atrial fibrillation (PCP-AF) patient	This meta-analysis of 12 randomized controlled trials (RCTs) analyzed the feasibility and safety of colchicine for the prevention of PCP-AF.	Colchicine can effectively prevent post-cardiac operative atrial fibrillation and relapse of atrial fibrillation after pulmonary vein isolation (PVI). However, colchicine can also increase the incidence of side effects, mainly gastrointestinal adverse events.
Imazio, M., et al (2014)	colchicine	post-pericardiotomy syndrome (POAF) patient	This was an investigator-initiated, double-blind, placebo-controlled, randomised clinical trial of 360 cardiac surgery patients randomised to receive colchicine or placebo, with the aim of determining the efficacy and safety of perioperative oral colchicine in reducing postpericardiotomy syndrome, postoperative AF, and postoperative pericardial or pleural effusions.	Among patients undergoing cardiac surgery, perioperative use of colchicine compared with placebo reduced the incidence of postpericardiotomy syndrome but not of postoperative AF or postoperative pericardial/pleural effusion. The increased risk of gastrointestinal adverse effects reduced the potential benefits of colchicine in this setting.
Conen, D., et al.(2023).	Colchicine	Non-Cardiac Surgery (MINS) patient	COP-AF was a randomised trial conducted at 45 sites in 11 countries, enrolling 3209 people. Assessing the Risk of Perioperative Atrial Fibrillation and Myocardial Injury After Non-Cardiac Surgery (MINS) with Oral	In patients undergoing major non-cardiac thoracic surgery, administration of colchicine did not significantly reduce the incidence of clinically important atrial fibrillation or MINS but increased the risk of mostly benign non-infectious diarrhoea.

Parent, S.,et al. (2024).	Colchicine	Rat model of aseptic pericarditis	Colchicine Evaluation of Amiodarone, Colchicine and EV Derived from Human Cardiac Source Cells for the Prevention of Postoperative AF in a Rat Model of Aseptic Pericarditis	In this study, both amiodarone and EVs prevented AF, whereas treatment with colchicine was ineffective.
Ahmed, A. S., et al.(2023).	colchicine 0.6 mg	Post-AF patient	PAPERS is a multicenter, prospective, randomized controlled study. Patients were randomized on the day of the procedure to receive no postprocedure prophylaxis (group A; standard of care arm) or colchicine 0.6 mg orally twice daily for 7 days starting immediately post-procedure (group B; study arm).The study aimed to quantify the risks and benefits associated with prophylactic use of colchicine to prevent pericarditis following AF ablation.	Prophylactic colchicine therapy initiated after the ablation procedure in patients with AF did not affect the incidence of post-ablation pericarditis and was associated with an increased incidence of gastrointestinal side effects.
Mohanty, S.,et al .(2023).	colchicine	AF patients undergoing first catheter ablation	Consecutive AF patients undergoing first catheter ablation were classified into 3 groups based on their colchicine use: Group 1: no	Colchicine therapy starting 7 days before to 1 month after the ablation procedure was associated with significantly lower risk of acute pericarditis and related hospitalization. In addition, paroxysmal AF

			colchicine; group 2: colchicine from 7 days before to 1 month after ablation; and group 3: colchicine from the day of the procedure to 1 month after.	patients receiving colchicine had a higher arrhythmia-free survival rate compared with those not receiving colchicine.
Lee, J. Z., et al.(2016).	colchicine	Post-AF patient	A meta-analysis of 3 randomised studies with 912 patients to assess the efficacy and safety of colchicine in postoperative atrial fibrillation	Current evidence suggests that colchicine therapy is efficacious for the prevention of post-operative AF
Viviano, A., Kanagasabay, R., & Zakkar, M. (2014).	corticosteroids	Perioperative patient	The article searched for 70 clinical trials or meta-analyses related to the treatment of atrial fibrillation with steroids to assess whether perioperative corticosteroid administration was associated with a reduced incidence of postoperative atrial fibrillation (POAF) in adult cardiac surgery	A single prophylactic moderate dose of corticosteroids significantly reduced the risk of POAF without a significant increase in morbidity or mortality
Losiggio, R., et al (2024)	corticosteroids	extracorporeal circulation (CPB) patient	A meta-analysis of 17 randomised controlled trials including patients under 65 years of age (paediatric and non-elderly) was designed to assess the effect of prophylactic corticosteroids in paediatric and non-	This meta-analysis of randomized trials highlights the potential benefits of corticosteroids on survival in cardiac surgery for patients younger than 65 years old.

			elderly adult cardiac surgery patients. The primary outcome was mortality at the longest available follow-up. Secondary outcomes included acute kidney injury, atrial fibrillation, myocardial injury, cerebrovascular events, and infections.	
Ho, K. M., & Tan, J. A. (2009)	corticosteroids	adult cardiac surgery requiring extracorporeal circulation	A meta-analysis of 3323 patients from 50 randomised controlled trials of adult cardiac surgery requiring extracorporeal circulation was included in the study to assess the effect of using different doses of corticosteroids on postoperative atrial fibrillation.	Corticosteroid prophylaxis reduced the risk of atrial fibrillation and length of stay in the intensive care unit, and low-dose corticosteroids were as effective as high-dose corticosteroids in reducing the risk of atrial fibrillation and duration of mechanical ventilation, but with fewer potential side effects in adult cardiac surgery.
Liu C,et al.2014,	glucocorticoids	postoperative atrial fibrillation	A total of 27 studies and 14,442 patients were included in the meta-analysis to assess the effect of different doses of glucocorticoids on postoperative atrial fibrillation.	The use of glucocorticoids reduces the incidence of POAF and is more effective at lower doses of glucocorticoids. Glucocorticoids may better suppress POAF in CABG.
Kim, Y. R.,et al(2015)	steroid	atrial fibrillation ablation.	This is a prospective study enrolling 138 patients who were randomly assigned to 2 groups (steroid group and control group) to	Periprocedural short-term moderate intensity steroid therapy reduces ER (≈3 months) after catheter ablation of atrial fibrillation. It is not effective in preventing late (3≈24 m) atrial fibrillation

			investigate the effect of steroids on early recurrence (ER) in the first 3 months (blanking period) after atrial fibrillation ablation.	recurrence.
Shiroshita-Takeshita, A., Brundel, B. J., Lavoie, J., & Nattel, S. (2006).	prednisolone, ibuprofen, cyclosporine-A	rapidly paced dogs	This study is an animal experiment to investigate the effect of anti-inflammatory drugs (prednisolone, ibuprofen) or cyclosporine-A (a calmodulin phosphatase inhibitor) on induced atrial fibrillation in rapidly paced dogs	Prednisone prevents the electrophysiological and atrial fibrillation-promoting effects of atrial tachycardia-remodeling, possibly by an anti-inflammatory action
Iskandar S, et al (2017)	prednisone	Radiofrequency ablation of pulmonary vein isolation patient	This study is a prospective trial in which a total of 60 patients with paroxysmal AF undergoing radiofrequency ablation were randomly assigned (1:1) to receive prednisone or placebo. The efficacy of oral prednisolone in improving outcomes of radiofrequency ablation of pulmonary vein isolation and its effect on inflammatory cytokines was assessed.	Although oral corticosteroids have significant effect in lowering certain cytokines, it did not impact the clinical outcomes of AF ablation.

Won H, 2013	hydrocortisone	radiofrequency catheter ablation (RFCA) patient	This is a prospective study of 89 patients with AF who underwent radiofrequency ablation and were treated with a single infusion of 100 mg hydrocortisone and 120 control patients to assess whether low-dose hydrocortisone is effective in preventing recurrence of AF after radiofrequency catheter ablation (RFCA).	Single bolus injection of low-dose hydrocortisone after AF ablation is not effective for preventing AF recurrence during the mid-term follow-up period.
Chai, T., et al. (2022).	corticosteroid	patients undergoing cardiac surgery with extracorporeal circulation.	This study was a meta-analysis of 88 RCTs involving 18,416 patients to assess the effect of different corticosteroid doses on clinical outcomes in patients undergoing cardiac surgery with extracorporeal circulation.	Low-dose corticosteroids associated with lower risk of atrial fibrillation
Zhang, H.,et al. (2022).	prednisone	atrial rapid pacing dog	This study is an animal experiment in which dogs were randomised into a sham-operated group, an atrial rapid pacing group (ATP group), an ATP + low-dose prednisone (ALP) group and an ATP + high-dose prednisone (AHP)	Prednisolone reduces the expression of type I collagen, type III collagen, α-smooth muscle actin, transforming growth factor-β1, and connective tissue growth factor, inhibits the formation of reactive oxygen species, and reduces the level of NADPH oxidase 2 protein, and prevents atrial fibrosis induced by rapid atrial

			group. To assess whether prednisone treatment prevents atrial fibrosis induced by atrial rapid pacing (ATP).	pacing.
Iwasaki, Y. K., et al. (2022)	dexamethasone	Sprague-Dawley rats	This is an animal study in which dexamethasone (DEX, 1.0 mg/kg) was injected subcutaneously into Sprague-Dawley rats. The cardiac ion channel genes were measured using an RNase protection assay at predetermined times (0, 1, 3, 6, 12, and 24 hours) after DEX injection to investigate whether glucocorticoids can alter the temporal profile of cardiac ion channel gene expression, leading to atrial arrhythmias.	These results implied that transient glucocorticoid-induced biochemical modification of cardiac ion channels might be one of the mechanisms underlying the stress-induced paroxysmal AF.
Liu, L., et al. (2021).	Corticosteroids	POAF	This study is a meta-analysis of 13 studies involving 803,0 patients aimed at assessing the efficacy and safety of corticosteroids in the prevention of POAF after cardiac surgery.	Corticosteroids Significantly Reduce Risk of POAF
Kuhn, E. W., et al (2021).	statin	peri-operative cardia patient	This is a meta-study that included 10 randomised controlled studies with a total of 3,468 subjects to	Pre-treatment with statins is associated with a formal reduction in the incidence of postoperative atrial fibrillation (AF), but given the associated

			assess the effect of statin intake on peri-operative cardiac outcomes	heterogeneity among the included studies, statin therapy is generally not recommended
Bockeria, O. L.,et al (2016).	statin	Post coronary artery bypass graft patients	A retrospective analysis of 206 patients undergoing isolated CABG was performed, and all patients were divided into two groups. The first group (nSt patients) consisted of patients who were not treated with statins before CABG (n=82). The second group (nSt patients) consisted of patients who received statins before CABG (n=124) to assess the role of statin therapy in the prevention of postoperative atrial fibrillation (POAF) after coronary artery bypass grafting (CABG) in patients with no prior atrial fibrillation.	Statin therapy carried out prior to the CABG is an effective approach to primary prevention of POAF in early postoperative period.
An, J., et al. (2017).	statin	Post coronary artery bypass graft patients	The effect of statins on early cardiac complications and inflammatory markers in coronary artery bypass grafting was assessed by meta-analysing data from 17 clinical studies with 2796 patients.	postoperative AF incidence is the biggest beneficiary of statin pretreatment in coronary artery bypass graft surgery. This effect is associated with beneficial modulation of systemic inflammatory markers.
Yan, P., et al. (2014).	statins	Patient after electrical	A meta-analysis of 5 clinical	Based on the currently available data,

		cardioversion (EC)	randomised controlled trials (involving 524 patients) was conducted to assess the effect of statins on the recurrence of AF after EC.	administration of statin agents, especially atorvastatin or rosuvastatin, is beneficial in lowering the frequency of AF recurrence after EC.
Allah, E. A., et al. (2019).	atorvastatin	postoperative atrial fibrillation	This study is a randomised controlled trial of 64 adult patients undergoing heart valve replacement surgery, randomised to an atorvastatin group and a placebo group, to evaluate the role of prophylactic high-dose atorvastatin in the prevention of postoperative atrial fibrillation (POAF), attenuation of the inflammatory response, and myocardial protection after valve replacement heart surgery.	Prophylactic use of high dose atorvastatin can decrease the incidence of POAF and attenuate the inflammatory process in adult patients undergoing isolated rheumatic cardiac valve replacement surgery.
Peña, J. M., et al. (2012).	rosuvastatin	Patients that low-density lipoprotein cholesterol <130 mg/dL and high-sensitivity C-reactive protein ≥2 mg/L	This was a retrospective study of 17 120 patients, randomising men and women with low-density lipoprotein cholesterol <130 mg/dL and high-sensitivity C-reactive protein ≥2 mg/L to receive either Rosuvastatin 20 mg daily or placebo Whether	Within the JUPITER trial cohort of individuals selected for underlying inflammation, increasing levels of high-sensitivity C-reactive protein were associated with an increased risk of incident AF and random allocation to rosuvastatin significantly reduced that risk.

			high-sensitivity C-reactive protein was associated with new-onset AF and whether treatment with Rosuvastatin was associated with a lower incidence of AF compared with placebo. 17 120	
Choi, S. E.,et al (2024).	statins	AF combined with ischaemic stroke	This is a retrospective cohort study of 20,902 patients with AF combined with ischaemic stroke to assess the benefits of statins in this population.	Patients with AF and recent IS, who received early statins, had a lower risk of recurrent stroke, death, and other cardiovascular outcomes including ICH, compared to those who did not.
Eun, M. Y., et al. (2020).	Statin	atrial fibrillation (AF)-related stroke	A meta-analysis of 12 studies evaluating the effect of statins on atrial fibrillation (AF)-related stroke.	Statin treatment of AF-related stroke may reduce all-cause mortality. CONCLUSION: Statins for AF-related stroke may reduce all-cause mortality and improve functional outcomes.
Moutzouri, E.,et al (2023).	statin	atrial fibrillation	This study investigated the association between statin use and lipid levels and the prevalence and progression of cerebral microbleeds (CMBs) in patients with AF by analysing data from Swiss-AF, a prospective cohort of patients with established AF.	In this prospective cohort of patients with AF, a population at increased hemorrhagic risk due to anticoagulation, the use of statins was not associated with an increased risk of CMBs.
Choi, K. H., et al.(2019).	statin	atrial fibrillation , acute ischaemic	The present study is a national, multicentre, cohort study of 2153	Statins, particularly high-intensity statins, could reduce the risk for NACCE in patients with acute

		stroke	patients with acute ischaemic stroke and atrial fibrillation to assess the effect of statin intensity on outcomes in these patients	ischemic stroke and atrial fibrillation
Huang, J. Y., et al. (2023).	statin	atrial fibrillation	This is a retrospective study that included patients with newly diagnosed AF from 2010 to 2018 who were statin users (n=23,239) and statin users (n=29,251) to assess the association between statin use and HF in patients with AF.	Sustained reduction in HF risk with statins
Bonano, J. C., et al. (2021).	simvastatin	total joint arthroplasty (TJA) patients	This study was a single-centre retrospective cohort of 231 primary total joint arthroplasty (TJA) patients (109 hip, 122 knee) designed to determine whether perioperative use of statins reduces the incidence of cardiac arrhythmias 90 days after surgery in patients with primary TJA.	Treating as few as 28 patients with perioperative simvastatin prevents one new cardiac arrhythmia within 90 days in statin-naïve patients undergoing TJA.
Oliveri, F., et al. (2022).	statin	cardiac surgery patient	A meta-analysis of randomised controlled trials (RCTs) between January 2006 and	statin pretreatment before cardiac surgery is not associated with a significant reduction in POAF occurrence.

			January 2022 assessing the effect of statin pretreatment on postoperative atrial fibrillation in patients undergoing cardiac surgery.	
Fiedler, L., et al (2021).	Statin	AF patient after CV.	This was a single-centre registry study including consecutive AF patients (n = 454) receiving CV, performing Cox regression models to assess AF recurrence, comparing patients with and without statins. The aim was to examine whether upstream statin therapy is associated with long-term recurrence of AF after CV.	Statin therapy is associated with a reduced risk of long-term AF recurrence after successful cardioversion.
Yu, Y., et al.(2022).	Statin	cardiac resynchronization therapy (CRT) patients	The study included 685 patients with CRT. Study patients were categorised into four groups based on AF status and statin use: non-AF/statin, non-AF/non-statin, AF/statin and AF/non-statin. Multivariate Cox proportional risk regression models were used to assess the independent and joint association of AF	AF was associated with poor prognosis, and statin use failed to improve the prognosis. Further analysis showed that statin therapy is ineffective in improving prognosis and fails to attenuate the adverse effects of AF.

			and statin therapy with poor prognosis in patients with CRT AF.	
Dong, S., et al. (2019).	Statin	stroke	Retrospective analysis of consecutive cases of ischaemic stroke occurring between 2011 and 2017, a total of 1878 patients, with propensity score matching and logistic regression analyses, was performed to assess the effect of low-dose statin therapy on stroke.	Low-Dose Statin Pretreatment Improves Initial Stroke Severity and Functional Outcomes at 90 Days
Zhang, J., et al.(2020).	rosuvastatin	AMI patients	An observational, retrospective cohort study was conducted in Jinan, China, in which 323 patients with AMI were recruited, and multivariate Cox and Kaplan-Meier analyses were used to assess independent factors and differences in AF and ischaemic events, as well as safety, across different doses of Rosuvastatin	Treatment with rosuvastatin can reduce the serum lipid level and improve cardiac function. Different dosages of rosuvastatin, age, smoking, drinking alcohol, and diabetes are independent risk factors for AF and ischemia events. The results suggested it is safe to use 20 mg rosuvastatin in the 12 months after hospital admission
Pastori, D., et al.(2021).	statin	non-valvular atrial fibrillation (AF)	The benefit of statin therapy on adverse cardiovascular outcomes in patients with non-valvular atrial	In AF patients, statin therapy was associated with a reduction in all-cause and cardiovascular mortality are reduced by 41 % and 25 %, respectively.

			fibrillation (AF) was assessed through a meta-analysis of 14 studies (post hoc analyses of 2 randomised clinical trials, 8 prospective and 4 retrospective analyses) involving 100,287 patients with AF, of whom 23,228 were taking statins.	
Qi, X., et al. (2023).	Omega-3 polyunsaturated fatty acids (PUFAs)	cardiovascular disease (CVD)	The article manually searched PubMed, EMBASE, the Cochrane Library, and Web of Science for eligible RCTs on the use of Omega-3 polyunsaturated fatty acids (PUFAs) for CVD, and included 116 eligible RCTs involving 498,3 people assessing the effects of Omega-3 supplementation on cardiovascular disease (CVD).	Omega-3 PUFA Supplementation Reduces the Risk of CV Death and Haemodialysis, Also Increases AF
Reiner, M. F., et al(2021).	omega-3 fatty acids (n-3 FA)	AF patients	EPA, docosahexaenoic acid (DHA), docosapentaenoic acid (DPA), and alpha-linolenic acid (ALA) were measured by gas chromatography in 1657 AF patients from the Swiss	EPA correlates inversely with the prevalence of ischemic brain infarcts, but not with markers of small vessel disease in patients with AF.

			Atrial Fibrillation Study. To assess whether omega-3 fatty acids (n-3 FA) correlate with stroke or cerebral small-vessel disease in patients with atrial fibrillation (AF).	
Rubanenko, O., et al(2023).	omega-3 index	patients admitted for CABG surgery.	This study included 158 patients admitted for CABG surgery. Postoperative atrial fibrillation (POAF) related inflammation, oxidative stress, fibrosis, myocardial dysfunction, ischemia and omega-3 index were analysed after coronary artery bypass grafting (CABG) in patients with coronary artery disease, divided into a group without POAF and a group with POAF.	The study showed that increased omega-3 index was associated with POAF in patients who underwent CABG.
Kalstad, A. A., et al.(2021).	Omega-3 Fatty Acids	Myocardial Infarction in the Elderly	The OMEMI trial (Omega-3 Fatty Acids in Myocardial Infarction in the Elderly) was an investigator-initiated, multicentre randomised clinical trial in which a total of 1,027 patients were randomised to a	n-3 polyunsaturated fatty acids did not reduce clinical events, including atrial fibrillation, in elderly patients with AMI.

			placebo group (corn oil) and an n-3 polyunsaturated fatty acid group, with the primary endpoints being the composite endpoints of non-fatal AMI, unplanned haemodialysis, stroke, all-cause mortality, and hospitalisation for heart failure at 2 years. The secondary end point was new-onset atrial fibrillation. The safe outcome was major bleeding. To assess the risk of subsequent cardiovascular events with 1.8 g n-3 PUFA in elderly patients with AMI.	
Nicholls, S. J.et al. (2020).	omega-3	high cardiovascular risk patients	This study was a double-blind, randomised, multicentre trial comparing omega-3 CA with corn oil in statin-treated participants with high cardiovascular risk, hypertriglyceridemia and low levels of high-density lipoprotein cholesterol (HDL-C). The primary efficacy indicator was a composite of cardiovascular	Among statin-treated patients at high cardiovascular risk, the addition of omega-3 CA, compared with corn oil, to usual background therapies resulted in no significant difference in a composite outcome of major adverse cardiovascular events

			death, nonfatal myocardial infarction, nonfatal stroke, coronary revascularisation, or unstable angina requiring hospitalisation. Assessing the Impact of omega-22 CA on Cardiovascular Outcomes	
Lombardi, M., et al (2021).	O3FA supplementation	Patients with Elevated Plasma Triglycerides and Elevated Risk of CV	This study is a meta-analysis that included 5 randomised controlled trials to assess whether supplementation with O3FA was associated with an increased risk of AF compared with placebo (corn oil).	O3FA supplementation associated with increased risk of AF
Gencer, B., et al (2021).	ω- fatty acids	AF	A total of seven randomised controlled trials were included in this study for meta-analysis. To assess the risk of ω- fatty acids and atrial fibrillation.	In RCTs examining cardiovascular outcomes, marine ω-3 supplementation was associated with an increased risk of AF. The risk appeared to be greater in trials testing >1 g/d.
Kowey, P. R., et al (2010).	omega-3 fatty acids	AF patients	This study was a prospective, randomised, double-blind, placebo-controlled, parallel-group, multicentre trial involving 663 US outpatients with a confirmed diagnosis of symptomatic paroxysmal (n =	Among participants with paroxysmal AF, 24-week treatment with prescription omega-3 compared with placebo did not reduce recurrent AF over 6 months.

			542) or persistent (n = 121) AF, no substantial structural heart disease, and normal sinus rhythm at baseline, to evaluate the safety and efficacy of prescribing omega-3 fatty acids (prescription omega-3s) to prevent recurrent symptomatic AF. Safety and Efficacy.	
Cao, H., et al.(2012).	omega-3 fatty acids	AF patients	Systematic Evaluation and Meta-Analysis to Assess the Efficacy of Omega-3 Fatty Acids in Preventing Recurrence of Atrial Fibrillation (AF) After Cardioversion by Searching Databases	omega-3 fatty acids did not have a significant effect on the prevention of AF recurrence after cardioversion, but the rate of AF recurrence was significantly lower in the subgroup of patients who took omega-3 fatty acids at least 4 weeks before cardioversion and continued to take them thereafter.
Ninio, D. M., et al. (2005).	tuna fish oil,sunflower oil	rabbits	Six-week-old rabbits were fed standard rabbit pellets supplemented with 5% tuna fish oil (n = 6) or supplemented with 5% sunflower oil (n = 6) for 12 weeks.,and using 3 rabbits raised on a standard diet as a control , applying rapid burst pacing during increases in intraatrial pressure to induce AF until	Incorporation of dietary omega-3 fatty acids into atrial tissue reduces stretch-induced susceptibility to AF.

			AF persisted (>6 minutes). The effect of dietary fish oil on the stretch-induced atrial fibrillation (AF) susceptibility rabbit model was evaluated.	
Sakabe, M., et al.(2007).	PUFA supplementation	atrial fibrillation dogs model	This paper investigates the role of oral PUFA supplementation in two dogs with atrial fibrillation, rapid atrial pacing-induced electrical remodelling (400 bpm for 1 week) and rapid ventricular pacing-induced structural remodelling associated with congestive heart failure (240 bpm for 2 weeks), and assesses the effect of PUFA supplementation on atrial fibrillation.	PUFAs suppress congestive heart failure-induced atrial structural remodeling and AF promotion but do not affect atrial tachycardia-induced electrical remodeling. The beneficial effects of PUFAs on structural remodeling, possibly related to prevention of mitogen-activated protein kinase activation,
Liao, J., et al.(2021).	IL-6 antibody	sterile pericarditis (SP) rats	SP was induced in rats by sprinkling the atria with sterile talcum powder. anti-rat IL-16 antibody (7.30 µg/kg) was administered intraperitoneally 6 min after recovery from anaesthesia. In vivo electrophysiology, ex vivo optical labelling, protein blotting and	IL-6-mediated-Ca2+ handling abnormalities in SP rats, especially RyR2-dysfunction, independent of IL-6-induced-fibrosis, early contribute to the development of POAF by increasing propensity for arrhythmogenic alternans.

			immunohistochemistry were performed to elucidate the mechanism of IL-6-mediated susceptibility to AF.	
Kondo, H., et al. (2018).	IL-10 antibody	8- to 10-week-old male CL57/B6 (wild-type) mice and IL-10 knockout mice	In this animal study, 8- to 10-week-old male CL57/B6 (wild-type) mice and IL-10 knockout mice were divided into 12-week HFD and 12-week normal diet (NFD) groups, respectively, to examine the effects of IL-10 on obesity-induced atrial inflammation, lipid deposition, fibrosis, and fibrillation induced by high-fat diet (HFD).	Our results highlight IL-10 treatment as a potential therapeutic approach to limit the progression of HFD-induced obesity-caused atrial fibrillation.
Fu, X. X.,et al (2015).	IL-17 antibody	SP mice	The present study is an animal experiment using sterile talcum powder to induce aseptic pericarditis (SP) in rats. AF was induced by transesophageal burst pacing. The levels of IL-17A in AF of SP rats were explored, as well as the effect of IL-17 antibodies on AF.	Elevated levels of atrial IL-17A are associated with the probability of developing AF. Treatment with anti-IL-17A monoclonal antibodies reduced atrial IL-17A levels, prolonged refractive error, and significantly inhibited the development of AF.
Cheng, T. Y., et al. (2024).	IL-33 ,anti-ST2 antibody	mice	The present study was an animal experiment in which telemetric ECG recordings, Masson's trichrome	IL-33 recombinant protein treatment promotes atrial remodeling through ST2 signaling. Blocking the IL-33/ST2 axis might be an innovative therapeutic

		staining and atrial immunohistochemical staining were performed in mice receiving tail vein injections of non-specific immunoglobulin (control), IL-33 and IL-33 combined with anti-ST2 antibody for 2 weeks, to analyse the effects of IL-33 and anti-ST2 antibody on arrhythmias.	approach for patients with atrial arrhythmia and elevated serum IL-33.

Reference

[1] Sinner, M. F., et al. (2014). B-type natriuretic peptide and C-reactive protein in the prediction of atrial fibrillation risk: the CHARGE-AF Consortium of community-based cohort studies. Europace : European pacing, arrhythmias, and cardiac electrophysiology : journal of the working groups on cardiac pacing, arrhythmias, and cardiac cellular electrophysiology of the European Society of Cardiology, *16*(10), pp.1426–1433. doi:10.1093/europace/euu175.

[2] Perelshtein B.O., et al. (2021). Measurements of immature platelet fraction and inflammatory markers in atrial fibrillation patients - Does persistency or ablation affect results?. International journal of laboratory hematology, *43*(4), pp.602–608. doi:10.1111/ijlh.13426.

[3] Sun, W., et al. (2019). C-reactive protein promotes inflammation through TLR4/NF-κB/TGF-β pathway in HL-1 cells. Bioscience reports, *39*(8), BSR20190888.doi:10.1042/BSR20190888.

[4] Gungor, B., et al. (2013). Assessment of interleukin-1 gene cluster polymorphisms in lone atrial fibrillation: new insight into the role of inflammation in atrial fibrillation. Pacing and clinical electrophysiology : PACE, 36(10), pp.1220–1227. doi:10.1111/pace.12182.

[5] Loeser, R. F., Erickson, E. A., & Long, D. L. (2008). Mitogen-activated protein kinases as therapeutic targets in osteoarthritis. Current opinion in rheumatology, 20(5), pp.581–586. doi:10.1097/BOR.0b013e3283090463.

[6] Monnerat, G., et al (2021). Author Correction: Macrophage-dependent IL-1β production induces

cardiac arrhythmias in diabetic mice. Nature communications, 12(1), p.7261. doi:10.1038/s41467-021-27508-w.
[7] Sharma, A., et al(2018). Use of Biomarkers to Predict Specific Causes of Death in Patients With A trial Fibrillation. Circulation, 138(16), pp.1666–1676.doi:10.1161/CIRCULATIONAHA.118.034125.
[8] Zhou, L., et al. (2007). IL-6 programs T(H)-17 cell differentiation by promoting sequential engagement of the IL-21 and IL-23 pathways. Nature immunology, 8(9), pp.967–974. doi:10.1038/ni1488.
[9] Dobrev, D., et al. (2019). Postoperative atrial fibrillation: mechanisms, manifestations and management. Nature reviews. Cardiology, 16(7), pp.417–436.doi:10.1038/s41569-019-0166-5.
[10] Kondo, H., et al. (2018). Interleukin 10 Treatment Ameliorates High-Fat Diet-Induced Inflammatory Atrial Remodeling and Fibrillation. Circulation. Arrhythmia and electrophysiology, 11(5), p.006040. doi:10.1161/CIRCEP.117.006040.
[11] Xu, L., et al. (2019). Interleukin-17A contributes to atrial fibrillation recurrence and left atrial reservoir function after catheter ablation. Polish archives of internal medicine, 129(6), pp.432–435. doi:10.20452/pamw.4489.
[12] Zhang,X.F.,et al.(2021).Changes and clinical significance of serum MMP-2, TIMP-2 and IL-17 in patients with different types of atrial fibrillation. Experimental and Laboratory Medicine,39(6),pp.1476-1478+1487.
[13] Miossec, P., & Kolls, J. K. (2012). Targeting IL-17 and TH17 cells in chronic inflammation. Nature reviews. Drug discovery, 11(10),pp 763–776.doi:10.1038/nrd3794.
[14] Guo,Y.Y.,et al.(2019).Study on the correlation between interleukin-18 and atrial fibrillation.Journal of Harbin Medical University,53(1):pp.47-52.
[15] Deng, H., et al. (2011). Role of tumor necrosis factor-alpha in the pathogenesis of atrial fibrillation. Chinese medical journal, 124(13), pp.1976–1982.
[16] Lakin, R., et al. (2023). Cardiomyocyte and endothelial cells play distinct roles in the tumour necrosis factor (TNF)-dependent atrial responses and increased atrial fibrillation vulnerability induced by endurance exercise training in mice. Cardiovascular research, 119(16), pp.2607–2622. doi:10.1093/cvr/cvad144.
[17] Liew, R., et al (2013). Role of tumor necrosis factor-α in the pathogenesis of atrial fibrosis and development of an arrhythmogenic substrate. Circulation journal : official journal of the Japanese Circulation Society, 77(5), pp.1171–1179. doi:10.1253/circj.cj-12-1155.
[18] Lu,Q. (2024).Clinical analysis of glucose-regulated protein 94 and transforming growth factor-β

1 in patients with atrial fibrillation. Bengbu Medical College.doi:10.26925/d.cnki.gbbyc.2023.000111.

[19] Stanciu, A. E., et al. (2018). The role of pro-fibrotic biomarkers in paroxysmal and persistent atrial fibrillation. Cytokine, 103,pp. 63–68.doi:10.1016/j.cyto.2017.12.026.

[20] Verheule, S., et al. (2004). Increased vulnerability to atrial fibrillation in transgenic mice with selective atrial fibrosis caused by overexpression of TGF-beta1. Circulation research, 94(11), pp.1458–1465. doi:10.1161/01.RES.0000129579.59664.9d.

[21] Xu,N.,et al. (2022).Correlation between NF-κB/TNF-α pathway and atrial fibrillation. Journal of Hainan Medical College,28(12),pp.898-903.doi:10.13210/j.cnki.jhmu.20220407.004.

[22] Liu, M., et al.(2013). Mitochondrial dysfunction causing cardiac sodium channel downregulation in cardiomyopathy. Journal of molecular and cellular cardiology, 54, pp.25–34. doi:10.1016/j.yjmcc.2012.10.011.

[23] Gao, G., & Dudley, S. C., Jr (2009). Redox regulation, NF-kappaB, and atrial fibrillation. Antioxidants & redox signaling, 11(9),pp. 2265–2277. doi:10.1089/ars.2009.2595.

[24] Chen, G., et al. (2018). Cardiomyocyte Inflammasome Signaling in Cardiomyopathies and Atrial Fibrillation: Mechanisms and Potential Therapeutic Implications. Frontiers in physiology, 9, p.1115. doi:10.3389/fphys.2018.01115.

[25] Yao, C., et al. (2018). Enhanced Cardiomyocyte NLRP3 Inflammasome Signaling Promotes Atri al Fibrillation. Circulation, 138(20),pp. 2227–2242. doi:10.1161/CIRCULATIONAHA.118.035202.

[26] Chen, G., et al. (2018). Cardiomyocyte Inflammasome Signaling in Cardiomyopathies and Atrial Fibrillation: Mechanisms and Potential Therapeutic Implications. Frontiers in physiology, 9, p.1115.doi:10.3389/fphys.2018.01115.

[27] Bai, J., et al. (2018). Ionic and cellular mechanisms underlying TBX5/PITX2 insufficiency-induced atrial fibrillation: Insights from mathematical models of human atrial cells. Scientific reports, 8(1), p.15642. doi:10.1038/s41598-018-33958-y.

[28] He, X., et al. (2011). Atrial fibrillation induces myocardial fibrosis through angiotensin II type 1 receptor-specific Arkadia-mediated downregulation of Smad7. Circulation research, 108(2), pp.164–175. doi:10.1161/CIRCRESAHA.110.234369.

[29] Khatib, R., et al. (2013). Blockade of the renin-angiotensin-aldosterone system (RAAS) for primary prevention of non-valvular atrial fibrillation: a systematic review and meta analysis of randomized controlled trials. International journal of cardiology, 165(1), pp.17–24.doi:10.1016/j.ijcard.2012.02.009.

[30] Wang, Q., et al. (2016). Perindopril for the prevention of atrial fibrillation recurrence after

radiofrequency catheter ablation: One-year experience. Heart rhythm, 13(10),pp.2040–2047. doi:10.1016/j.hrthm.2016.06.039.
[31] Sun, Y. L., et al. (2018). Valsartan reduced the vulnerability to atrial fibrillation by preventing action potential prolongation and conduction slowing in castrated male mice. Journal of cardiovascular electrophysiology, *29*(10), pp.1436–1443. doi:10.1111/jce.13697.
[32] Li, S. N., et al. (2022). Sacubitril/Valsartan Decreases Atrial Fibrillation Susceptibility by Inhibiting Angiotensin II-Induced Atrial Fibrosis Through p-Smad2/3, p-JNK, and p-p38 Signaling Pathways. Journal of cardiovascular translational research, *15*(1), 131–142.doi:10.1007/s12265-021-10137-5.
[33] Huang, C. Y., et al. (2018). Renin-angiotensin-aldosterone blockade reduces atrial fibrillation in hypertrophic cardiomyopathy. Heart (British Cardiac Society), 104(15),pp.1276–1283. doi:10.1136/heartjnl-2017-312573.
[34] Chaugai, S., et al. (2016). Effect of RAAS blockers on adverse clinical outcomes in high CVD risk subjects with atrial fibrillation: A meta-analysis and systematic review of randomized controlled trials. Medicine, 95(26), P.4059. doi:10.1097/MD.0000000000004059.
[35] Jibrini, M. B., Molnar, J., & Arora, R. R. (2008). Prevention of atrial fibrillation by way of abrogation of the renin-angiotensin system: a systematic review and meta-analysis. American journal of therapeutics, 15(1), pp.36–43.doi:10.1097/MJT.0b013e31804beb59.
[36] Li, T. J., et al. (2013). Renin-angiotensin system inhibitors for prevention of recurrent atrial fibrillation: a meta-analysis. International journal of clinical practice, 67(6),pp.536–543. doi:10.1111/ijcp.12063.
[37] Dabrowski, R., et al. (2010). Effect of combined spironolactone-β-blocker ± enalapril treatment on occurrence of symptomatic atrial fibrillation episodes in patients with a history of paroxysmal atrial fibrillation (SPIR-AF study). The American journal of cardiology, 106(11), pp.1609–1614.doi:10.1016/j.amjcard.2010.07.037.
[38] Ito, Y.,et al. (2013). Effect of eplerenone on maintenance of sinus rhythm after catheter ablation in patients with long-standing persistent atrial fibrillation. The American journal of cardiology, 111(7),pp. 1012–1018. doi:10.1016/j.amjcard.2012.12.020.
[39] Li, D., et al. (2001). Effects of angiotensin-converting enzyme inhibition on the development of the atrial fibrillation substrate in dogs with ventricular tachypacing-induced congestive heart failure. Circulation, 104(21), pp.2608–2614.doi:10.1161/hc4601.099402.
[40] Roşianu, Ş. H., et al. (2013). Inflammatory markers in paroxysmal atrial fibrillation and the

protective role of renin-angiotensin-aldosterone system inhibitors. Clujul medical (1957), *86*(3), pp.217–221.

[41] Boldt, A., et al. (2006). ACE-inhibitor treatment attenuates atrial structural remodeling in patients with lone chronic atrial fibrillation. Basic research in cardiology, 101(3), pp.261–267. doi:10.1007/s00395-005-0571-2.

[42] Zhao, J., et al. (2020). The Effect of Renin-Angiotensin System Inhibitors on the Recurrence of Atrial Fibrillation After Catheter Ablation. International heart journal, 61(6), pp.1174–1182. doi:10.1536/ihj.20-346.

[43] Liu, T., et al. (2016). Mineralocorticoid receptor antagonists and atrial fibrillation: a meta-analysis. Europace : European pacing, arrhythmias, and cardiac electrophysiology : journal of the working groups on cardiac pacing, arrhythmias, and cardiac cellular electrophysiology of the European Society of Cardiology, *18*(5), pp.672–678.doi:10.1093/europace/euv366.

[44] Miceli, A., et al. (2009). Effects of angiotensin-converting enzyme inhibitor therapy on clinical outcome in patients undergoing coronary artery bypass grafting. Journal of the American College of Cardiology, 54(19), pp.1778–1784. doi:10.1016/j.jacc.2009.07.008.

[45] Wang, W. W., Zhang, F. L., Chen, J. H., Chen, X. H., Fu, F. Y., Tang, M. R., & Chen, L. L. (2015). Telmisartan reduces atrial arrhythmia susceptibility through the regulation of RAS-ERK and PI3K-Akt-eNOS pathways in spontaneously hypertensive rats. *Canadian journal of physiology and pharmacology*, *93*(8), 657–665. .doi:10.1139/cjpp-2014-0416.

[46] Zhang,F.H,et al. (2019).Effects of angiotensin-(1-7) on atrial remodelling and p38MAPK protein expression in fast atrial pacing dogs. Journal of Tianjin Medical University (05),pp 450-454.

[47] Cao,Y. (2021). Intervention of angiotensin 1-7 on tumour necrosis factor-a stimulated inflammatory signalling pathway of MKK/P38MAPKs in mouse HL-1 cells (Master's thesis, Tianjin Medical University). Master's degree. doi:10.27366/d.cnki.gtyku.2021.000641.

[48] Bai,L. (2021). Effect of Ang1-7 on P38MAPK pathway and inflammation in HL-1 cells (Master's degree thesis, Tianjin Medical University). Master doi:10.27366/d.cnki.gtyku.2021.000913.

[49] Nakashima, H., et al. (2000). Angiotensin II antagonist prevents electrical remodeling in atrial fibrillation. Circulation, 101(22), pp.2612–2617. doi:10.1161/01.cir.101.22.2612.

[50] Liu,E.et al.(2011).Enalapril,irbesartan,and angiotensin-(1-7)prevent atrial tachycardia-induced ionic remodeling.Int J Car diol,146(3),pp364-370.

[51] Saygili, E., et al. (2007). Losartan prevents stretch-induced electrical remodeling in cultured

atrial neonatal myocytes. American journal of physiology. Heart and circulatory physiology, 292(6), pp.2898–2905.doi:10.1152/ajpheart.00546.2006.
[52] Takemoto, Y., et al. (2017). Eplerenone Reduces Atrial Fibrillation Burden Without Preventing Atrial Electrical Remodeling. Journal of the American College of Cardiology, 70(23), pp.2893–2905. doi:10.1016/j.jacc.2017.10.014.
[53] Yang, S. S., et al. (2008). Effects of spironolactone on electrical and structural remodeling of atrium in congestive heart failure dogs. Chinese medical journal, 121(1), 38–42.
[54] Ocal, A. G., et al. (2020). Colchicine's Effects on Electrocardiographic Parameters in Newly Diagnosed Familial Mediterranean Fever Patients : Colchicine may have Favourable Effects on Parameters Related to Ventricular Arrhythmias in New Diagnosed Familial Mediterranean Fever. Wirkungen von Colchizin auf EKG-Parameter bei Patienten mit neu diagnostiziertem familiärem Mittelmeerfieber : Positive Wirkungen von Colchizin auf Parameter ventrikulärer Arrhythmien bei neu diagnostiziertem familiärem Mittelmeerfieber. Zeitschrift fur Rheumatologie, 79(2), pp.210–215. doi:10.1007/s00393-019-0642-7.
[55] Wang, X., et al. (2022). Colchicine for Prevention of Post-Cardiac Surgery and Post-Pulmonary Vein Isolation Atrial Fibrillation: A Meta-Analysis. Reviews in cardiovascular medicine, 23(12), 387.doi:10.31083/j.rcm2312387.
[56] Imazio, M., et al (2014). Colchicine for prevention of postpericardiotomy syndrome and postoperative atrial fibrillation: the COPPS-2 randomized clinical trial. JAMA, 312(10), 1016–1023. doi:10.1001/jama.2014.11026.
[57] Conen, D.,et al (2023). Effect of colchicine on perioperative atrial fibrillation and myocardial injury after non-cardiac surgery in patients undergoing major thoracic surgery (COP-AF): an international randomised trial. Lancet (London, England), 402(10413),pp.1627–1635. doi:10.1016/S0140-6736(23)01689-6.
[58] Parent, S.,et al. (2024). Atrial Fibrosis and Inflammation in Postoperative Atrial Fibrillation: Comparative Effects of Amiodarone, Colchicine, or Exosomes. JACC. Clinical electrophysiology, 10(6), pp.1037–1049. doi:10.1016/j.jacep.2024.02.019.
[59] Ahmed, A. S.,et al. (2023). Prophylactic Colchicine After Radiofrequency Ablation of Atrial Fibrillation: The PAPERS Study. JACC. Clinical electrophysiology, 9(7 Pt 2), pp.1060–1066. doi:10.1016/j.jacep.2023.02.003.
[60] Mohanty, S., et al. (2023). Impact of Colchicine Monotherapy on the Risk of Acute Pericarditis Following Atrial Fibrillation Ablation. JACC. Clinical electrophysiology, 9(7 Pt 2), pp.1051–1059.

doi:10.1016/j.jacep.2023.01.037.

[61] Lee, J. Z.,et al. (2016). Colchicine for Prevention of Post-Operative Atrial Fibrillation: A Meta-Analysis. JACC. Clinical electrophysiology, 2(1),pp. 78–85. doi:10.1016/j.jacep.2015.09.016.

[62] Viviano, A., Kanagasabay, R., & Zakkar, M. (2014). Is perioperative corticosteroid administration associated with a reduced incidence of postoperative atrial fibrillation in adult cardiac surgery?. Interactive cardiovascular and thoracic surgery, 18(2), pp.225–229. doi:10.1093/icvts/ivt486.

[63] Losiggio, R., et al (2024). The Effects of Corticosteroids on Survival in Pediatric and Nonelderly Adult Patients Undergoing Cardiac Surgery: A Meta-analysis of Randomized Studies. Journal of cardiothoracic and vascular anesthesia, 38(11), pp.2783–2791.doi:10.1053/j.jvca.2024.07.001.

[64] Ho, K. M., & Tan, J. A. (2009). Benefits and risks of corticosteroid prophylaxis in adult cardiac surgery: a dose-response meta-analysis. Circulation, 119(14), pp.1853–1866. doi:10.1161/CIRCULATIONAHA.108.848218.

[65] Liu, C., et al. (2014). The efficacy of glucocorticoids for the prevention of atrial fibrillation, or length of intensive care unite or hospital stay after cardiac surgery: a meta-analysis. Cardiovascular therapeutics, 32(3), 89–96. doi:10.1111/1755-5922.12062.

[66] Kim, Y. R., et al. (2015). Effect of Short-Term Steroid Therapy on Early Recurrence During the Blanking Period After Catheter Ablation of Atrial Fibrillation. Circulation. Arrhythmia and electrophysiology, *8*(6), 1366–1372. doi:10.1161/CIRCEP.115.002957.

[67] Shiroshita-Takeshita, A., Brundel, B. J., Lavoie, J., & Nattel, S. (2006). Prednisone prevents atrial fibrillation promotion by atrial tachycardia remodeling in dogs. Cardiovascular research, 69(4), PP.865–875.doi:10.1016/j.cardiores.2005.11.028

[68] Iskandar, S., et al. (2017). Use of Oral Steroid and its Effects on Atrial Fibrillation Recurrence and Inflammatory Cytokines Post Ablation - The Steroid AF Study. Journal of atrial fibrillation, 9(5), 1604. doi:10.4022/jafib.1604.

[69] Won, H., et al. (2013). Effect of a single bolus injection of low-dose hydrocortisone for prevention of atrial fibrillation recurrence after radiofrequency catheter ablation. Circulation journal : official journal of the Japanese Circulation Society, 77(1), pp.53–59.doi:10.1253/circj.cj-12-0728.

[70] Chai, T., et al. (2022). Meta-Analysis: Shouldn't Prophylactic Corticosteroids be Administered During Cardiac Surgery with Cardiopulmonary Bypass?. Frontiers in surgery, 9, p.832205. doi:10.3389/fsurg.2022.832205.

[71] Zhang, H.,et al. (2022). Prednisone Ameliorates Atrial Inflammation and Fibrosis in Atrial

Tachypacing Dogs. International heart journal, 63(2), pp.347–355. doi:10.1536/ihj.21-249
[72] Iwasaki, Y. K., et al. (2022). Glucocorticoid Induces Atrial Arrhythmogenesis via Modification of Ion Channel Gene Expression in Rats. International heart journal, 63(2),pp.375–383. doi:10.1536/ihj.21-677.
[73] Liu, L.,et al. (2021). Effects of corticosteroids on new-onset atrial fibrillation after cardiac surgery: A meta-analysis of randomized controlled trials. Medicine, 100(11), p.25130.doi:10.1097/MD.0000000000025130.
[74] Kuhn, E. W., et al. (2021). Preoperative Statin Therapy for Atrial Fibrillation and Renal Failure after Cardiac Surgery. The Thoracic and cardiovascular surgeon, 69(2), pp.141–147. doi:10.1055/s-0040-1710322.
[75] Bockeria, O. L., et al. (2016). Statin therapy in the primary prevention of early atrial fibrillation after coronary artery bypass grafting. Indian heart journal, 68(6), pp.792–797.doi:10.1016/j.ihj.2016.04.002.
[76] An, J., et al (2017). Preoperative statins as modifiers of cardiac and inflammatory outcomes following coronary artery bypass graft surgery: a meta-analysis. Interactive cardiovascular and thoracic surgery, 25(6), pp.958–965.doi:10.1093/icvts/ivx172.
[77] Yan, P., et al. (2014). Statin therapy decreased the recurrence frequency of atrial fibrillation after electrical cardioversion: a meta-analysis. Medical science monitor : international medical journal of experimental and clinical research, 20, pp.2753–2758. doi:10.12659/MSM.891049.
[78] Allah, E. A., et al. (2019). Could Short-Term Perioperative High-Dose Atorvastatin Offer Antiarrhythmic and Cardio-Protective Effects in Rheumatic Valve Replacement Surgery?. Journal of cardiothoracic and vascular anesthesia, 33(12),pp. 3340–3347. doi:10.1053/j.jvca.2019.05.013
[79] Peña, J. M., et al. (2012). High-sensitivity C-reactive protein, statin therapy, and risks of atrial fibrillation: an exploratory analysis of the JUPITER trial. European heart journal, 33(4), pp.531–537.doi:10.1093/eurheartj/ehr460,
[80] Choi, S. E.,et al. (2024). Early statin use is associated with improved survival and cardiovascular outcomes in patients with atrial fibrillation and recent ischaemic stroke: A propensity-matched analysis of a global federated health database. European stroke journal,p.23969873241274213. Advance online publication. doi:10.1177/23969873241274213.
[81] Eun, M. Y.,et al. (2020). Statin Effects in Atrial Fibrillation-Related Stroke: A Systematic Review and Meta-Analysis. Frontiers in neurology, 11, p.589684.doi:10.3389/fneur.2020.589684.
[82] Moutzouri, E.,et al. (2023). Association of statin use and lipid levels with cerebral microbleeds

and intracranial hemorrhage in patients with atrial fibrillation: A prospective cohort study. International journal of stroke : official journal of the International Stroke Society, 18(10), pp.1219–1227. doi:10.1177/17474930231181010.

[83] Choi, K. H., et al. (2019). Effect of Statin Therapy on Outcomes of Patients With Acute Ischemic Stroke and Atrial Fibrillation. Journal of the American Heart Association, 8(24), p.013941.doi:10.1161/JAHA.119.013941.

[84] Huang, J. Y., et al. (2023). Statin Therapy Is Associated With a Lower Risk of Heart Failure in Patients With Atrial Fibrillation: A Population-Based Study. Journal of the American Heart Association, 12(23), p032378. doi:10.1161/JAHA.123.032378.

[85] Bonano, J. C., et al. (2021). Perioperative Statin Use May Reduce Postoperative Arrhythmia Rates After Total Joint Arthroplasty. The Journal of arthroplasty, 36(10), pp.3401–3405.doi:10.1016/j.arth.2021.05.022.

[86] Oliveri, F., et al. (2022). Statin and Postcardiac Surgery Atrial Fibrillation Prevention: A Systematic Review and Meta-Analysis. Journal of cardiovascular pharmacology, 80(2), pp.180–186. doi:10.1097/FJC.0000000000001294.

[87] Fiedler, L., et al. (2021). Upstream Statin Therapy and Long-Term Recurrence of Atrial Fibrillation after Cardioversion: A Propensity-Matched Analysis. Journal of clinical medicine, 10(4), p.807. doi:10.3390/jcm10040807.

[88] Yu, Y., et al. (2022). Independent and Joint Association of Statin Therapy with Adverse Outcomes in Heart Failure Patients with Atrial Fibrillation Treated with Cardiac Resynchronization Therapy. Journal of inflammation research, 15, pp.6645–6656.doi:10.2147/JIR.S390127.

[89] Dong, S., et al. (2019). Low-dose statin pretreatment reduces stroke severity and improves functional outcomes. Journal of neurology, 266(12),pp. 2970–2978.doi:10.1007/s00415-019-09520-9.

[90] Zhang, J.,et al (2020). Effect of 2 Different Dosages of Rosuvastatin on Prognosis of Acute Myocardial Infarction Patients with New-Onset Atrial Fibrillation in Jinan, China. Medical science monitor : international medical journal of experimental and clinical research, 26, p.925666.doi:10.12659/MSM.925666.

[91] Pastori, D., et al. (2021). Statin use and mortality in atrial fibrillation: A systematic review and meta-analysis of 100,287 patients. Pharmacological research, 165,p.105418. doi:10.1016/j.phrs.2021.105418.

[92] Qi, X., et al. (2023). Omega-3 Polyunsaturated Fatty Acids Supplements and Cardiovascular Disease Outcome: A Systematic Review and Meta-Analysis on Randomized Controlled

Trials. Reviews in cardiovascular medicine, 24(1), p.24.doi:10.31083/j.rcm2401024.
[93] Reiner, M. F., et al. (2021). The Omega-3 Fatty Acid Eicosapentaenoic Acid (EPA) Correlates Inversely with Ischemic Brain Infarcts in Patients with Atrial Fibrillation. Nutrients, 13(2), 651.doi:10.3390/nu13020651.
[94] Rubanenko, O., Rubanenko, A., & Davydkin, I. (2023). Comprehensive Analysis of Factors Associated with New Episode of Postoperative Atrial Fibrillation after Coronary Artery Bypass Graft Surgery. Life (Basel, Switzerland), 13(10), p.2035. doi:10.3390/life13102035.
[95] Kalstad, A. A., et al (2021). Effects of n-3 Fatty Acid Supplements in Elderly Patients After Myocardial Infarction: A Randomized, Controlled Trial. Circulation, 143(6), pp.528–539. doi:10.1161/CIRCULATIONAHA.120.052209
[96] Nicholls, S. J., et al. (2020). Effect of High-Dose Omega-3 Fatty Acids vs Corn Oil on Major Adverse Cardiovascular Events in Patients at High Cardiovascular Risk: The STRENGTH Randomized Clinical Trial. JAMA, 324(22),pp.2268–2280. doi:10.1001/jama.2020.22258.
[97] Lombardi, M., et al. (2021). Omega-3 fatty acids supplementation and risk of atrial fibrillation: an updated meta-analysis of randomized controlled trials. European heart journal. Cardiovascular pharmacotherapy, 7(4), pp.69–70. doi:10.1093/ehjcvp/pvab008.
[98] Gencer, B., et al. (2021). Effect of Long-Term Marine ω-3 Fatty Acids Supplementation on the Risk of Atrial Fibrillation in Randomized Controlled Trials of Cardiovascular Outcomes: A Systematic Review and Meta-Analysis. Circulation, 144(25), pp.1981–1990.doi:10.1161/CIRCULATIONAHA.121.055654.
[99] Kowey, P. R., et al. (2010). Efficacy and safety of prescription omega-3 fatty acids for the prevention of recurrent symptomatic atrial fibrillation: a randomized controlled trial. JAMA, 304(21), pp.2363–2372.doi:10.1001/jama.2010.1735
[100] Cao, H., et al.. (2012). Omega-3 fatty acids in the prevention of atrial fibrillation recurrences after cardioversion: a meta-analysis of randomized controlled trials. Internal medicine (Tokyo, Japan), 51(18), pp.2503–2508. doi:10.2169/internalmedicine.51.7714.
[101] Ninio, D. M., et al. (2005). Dietary fish oil protects against stretch-induced vulnerability to atrial fibrillation in a rabbit model. Journal of cardiovascular electrophysiology, 16(11), pp.1189–1194.doi:10.1111/j.1540-8167.2005.50007.x.
[102] Sakabe, M., et al (2007). Omega-3 polyunsaturated fatty acids prevent atrial fibrillation associated with heart failure but not atrial tachycardia remodeling. Circulation, 116(19), pp.2101–2109. doi:10.1161/CIRCULATIONAHA.107.704759.

[103] Liao, J., et al. (2021). Interleukin-6-Mediated-Ca2+ Handling Abnormalities Contributes to Atrial Fibrillation in Sterile Pericarditis Rats. Frontiers in immunology, 12, p.758157.doi:10.3389/fimmu.2021.758157.

[104] Kondo, H., et al. (2018). Interleukin 10 Treatment Ameliorates High-Fat Diet-Induced Inflammatory Atrial Remodeling and Fibrillation. Circulation. Arrhythmia and electrophysiology, 11(5), p.006040.doi:10.1161/CIRCEP.117.006040.

[105] Fu, X. X., et al. (2015). Interleukin-17A contributes to the development of post-operative atrial fibrillation by regulating inflammation and fibrosis in rats with sterile pericarditis. International journal of molecular medicine, *36*(1), pp.83–92.doi:10.3892/ijmm.2015.2204.

[106] Cheng, T. Y., et al. (2024). Interleukin-33/ST2 axis involvement in atrial remodeling and arrhythmogenesis. Translational research : the journal of laboratory and clinical medicine, 268, 1–12. doi:10.1016/j.trsl.2024.01.006.

2 Supplementary Figures

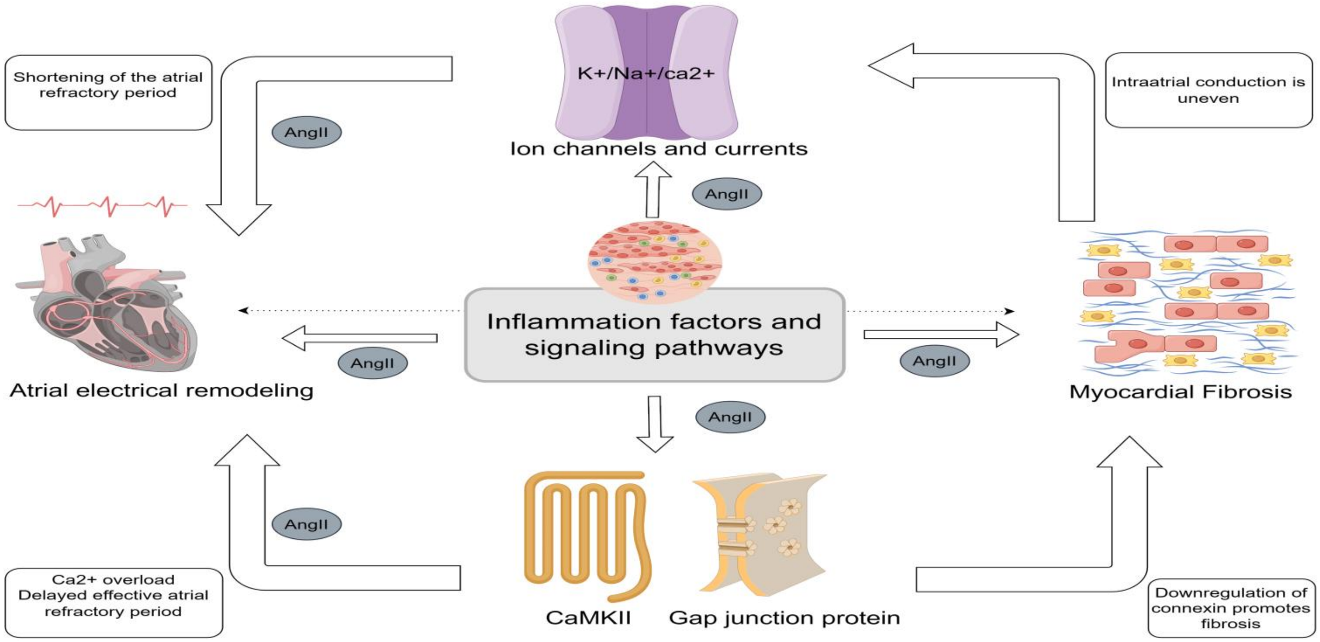

Supplementary Figure 1. Central illustration（By Figdraw）. Central role of inflammation in Angiotensin II-Induced Atrial Fibrillation. Inflammatory factors and inflammatory signaling pathways can contribute to atrial fibrillation by modulating ion channels and ion currents, calmodulin, and gap junction proteins to promote shortening and delaying of the effective atrial opriod. Myocardial fibrosis is the result of the co-regulation of Angiotensin II and multiple inflammatory signaling pathways. Fibrosis can lead to heterogeneous electrical signaling in the atria, affecting ion channels and currents and contributing to the occurrence of electrical remodeling.

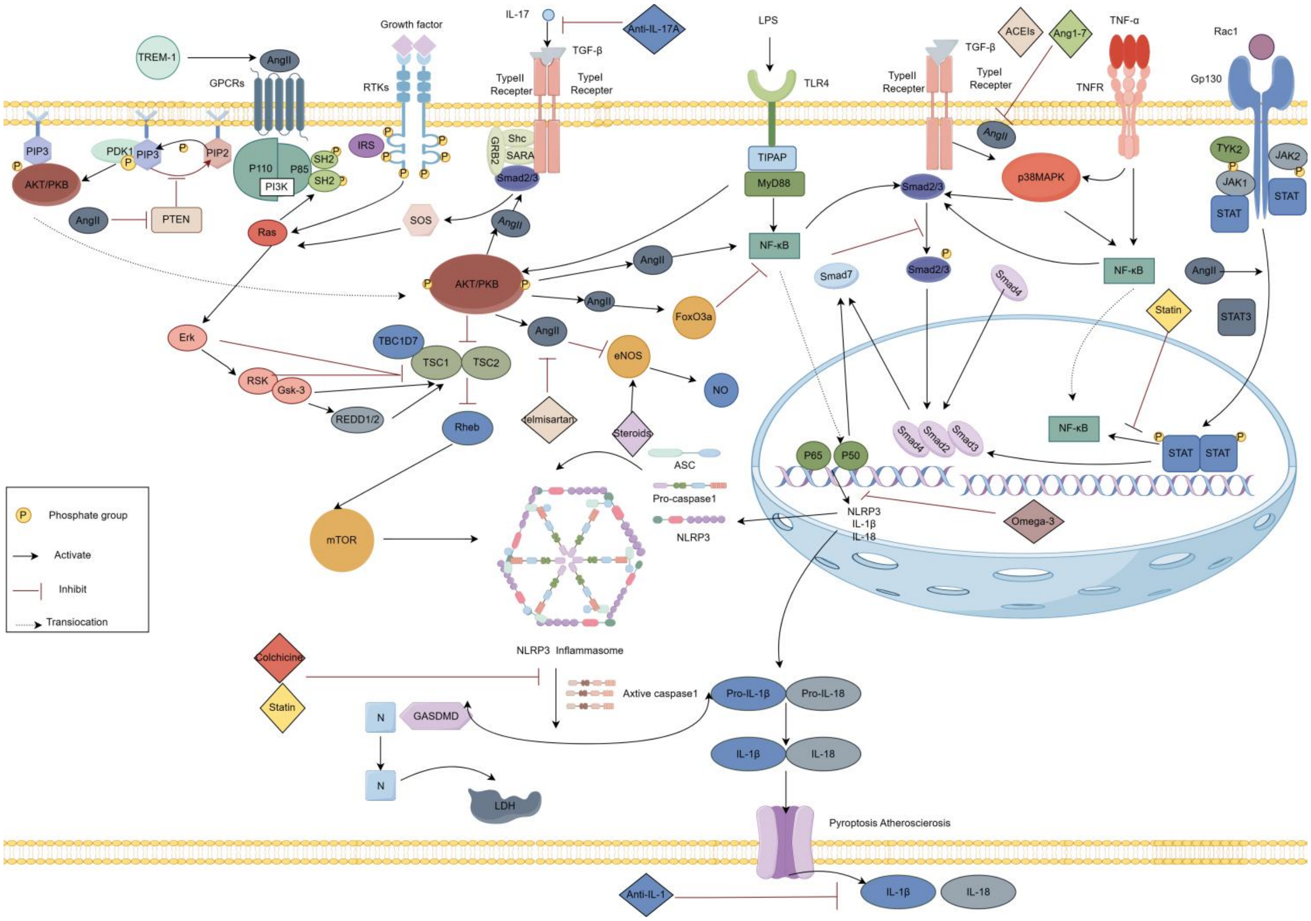

Supplementary Figure 2. Inflammatory crosstalk and anti-inflammatory mechanisms in the angiotensin II-induced fibrosis pathway.(By Figdraw)

AngII mediates the PI3K/AKT signaling pathway and participates in upstream PTEN, downstream TGF-β, m TOR, FoxO3a and e NOS signaling pathways, as well as the P38MAPK, JAK/STAT3 and NF-κB signaling pathways, and NF-κB further activates the NLRP3 inflammatory vesicles, releasing IL-1β, IL-18.Anti-inflammatory drugs act on the corresponding inflammatory pathways.

TLR,Toll-like Receptor;IGF1,Insulin-like growth factor 1 ;TNF，Tumor necrosis factor；TGF-β，Transforming growth factor-β；IL，Interleukins；PI3K,Phosphatidylinositol 3-kinase;PIP3,Phosphatidylinositol Trisphosphate;PDK,Phosphatidylinositol-dependent kinase;FoxO3a,Forkhead box O3;mTOR,The Mechanistic Target of Rapamycin;e NOS,Endothelial nitric oxide synthas;JAK,Janus tyrosine Kinase;STAT,Signal Transducer and Activator ofTranscription;NF-κB，Nuclear factor kappa-B protein;NLRP3，NOD-, LRR-, and pyrin domain-containing protein 3.
